# LRRK2 kinase activity regulates GCase level and enzymatic activity differently depending on cell type in Parkinson’s disease

**DOI:** 10.1038/s41531-022-00354-3

**Published:** 2022-07-19

**Authors:** Maria Kedariti, Emanuele Frattini, Pascale Baden, Susanna Cogo, Laura Civiero, Elena Ziviani, Gianluca Zilio, Federico Bertoli, Massimo Aureli, Alice Kaganovich, Mark R. Cookson, Leonidas Stefanis, Matthew Surface, Michela Deleidi, Alessio Di Fonzo, Roy N. Alcalay, Hardy Rideout, Elisa Greggio, Nicoletta Plotegher

**Affiliations:** 1 Division of Basic Neurosciences, Biomedical Research Foundation of the Academy of Athens, Athens, Greece; 2 Neurology Unit, Foundation IRCCS Ca’ Granda Ospedale Maggiore Policlinico, Milan, Italy; 3 Dino Ferrari Center, Neuroscience Section, Department of Pathophysiology and Transplantation, University of Milan, Milan, Italy; 4 German Center for Neurodegenerative Diseases (DZNE), Tübingen, 72076 Germany; 5 Department of Biology, University of Padova, Padova, Italy; 6 IRCCS San Camillo Hospital, Venice, Italy; 7 Centro Studi per la Neurodegenerazione (CESNE), University of Padova, Padova, Italy; 8 Department of of Medical Biotechnology and Translational Medicine, University of Milan, Milan, Italy; 9 Laboratory of Neurogenetics, NIA, Bethesda, USA; 10 Department of Neurology, University of Athens Medical School, Athens, Greece; 11 Department of Neurology, Columbia University Irving Medical Center, New York, USA; 12 Present Address: School of Biological Sciences, University of Reading, Reading, UK

**Keywords:** Cellular neuroscience, Enzymes

## Abstract

Leucine-rich repeat kinase 2 (LRRK2) is a kinase involved in different cellular functions, including autophagy, endolysosomal pathways, and immune function. Mutations in LRRK2 cause autosomal-dominant forms of Parkinson’s disease (PD). Heterozygous mutations in GBA1, the gene encoding the lysosomal enzyme glucocerebrosidase (GCase), are the most common genetic risk factors for PD. Moreover, GCase function is altered in idiopathic PD and in other genetic forms of the disease. Recent work suggests that LRRK2 kinase activity can regulate GCase function. However, both a positive and a negative correlation have been described. To gain insights into the impact of LRRK2 on GCase, we performed a comprehensive analysis of GCase levels and activity in complementary LRRK2 models, including (i) LRRK2 G2019S knock in (GSKI) mice, (ii) peripheral blood mononuclear cell (PBMCs), plasma, and fibroblasts from PD patients carrying LRRK2 G2019S mutation, (iii) patient iPSCs-derived neurons; (iv) endogenous and overexpressed cell models. In some of these models we found a positive correlation between the activities of LRRK2 and GCase, which was further confirmed in cell lines with genetic and pharmacological manipulation of LRRK2 kinase activity. GCase protein level is reduced in GSKI brain tissues and in G2019S iPSCs-derived neurons, but increased in fibroblasts and PBMCs from patients, suggesting cell-type-specific effects. Overall, our study indicates that LRRK2 kinase activity affects both the levels and the catalytic activity of GCase in a cell-type-specific manner, with important implications in the context of therapeutic application of LRRK2 inhibitors in GBA1-linked and idiopathic PD.

## Introduction

Mutations in *LRRK2* cause autosomal dominant Parkinson’s disease (PD) with age- and mutation-dependent penetrance^1–3^, whereas heterozygous mutations in *GBA1* are the most common genetic risk factors for PD and the cause of the lysosomal storage disorder Gaucher disease when present in homozygosis^4,5^. Leucine-rich repeat kinase 2 (LRRK2) is a large, multi-domain protein with two enzymatic domains, a Ser/Thr kinase domain and a small GTPase domain (ROC), where the bulk of the pathogenic PD-linked mutations are located. While its full range of cellular functions has yet to be characterized, it has been robustly associated with endo-lysosomal pathways and vesicular trafficking (reviewed in Bonet-Ponce and Cookson, 2021^6^). These activities are likely mediated by its phosphorylation of multiple members of the Rab GTPase family, which is increased in the context of the disease-linked mutations^7^, and potentially also in cases of PD not linked to mutations in *LRRK2*^8^.

The main function of the lysosomal enzyme glucocerebrosidase (GCase) is to hydrolyze glucosylceramide and glucosylsphingosine to glucose and either ceramide or sphingosine, respectively; and most of the mutations in *GBA1* associated with PD risk reduce the activity of GCase^4,9^. High levels of α-synuclein, another protein mutated in PD and the major component of Lewy bodies, inhibit autophagic flux and the lysosomal activity of GCase^10^. GCase activity has been shown to be reduced also in peripheral monocyte extracts from PD patients without mutations in *GBA1*^11,12^ and in PD brains^13^, overall suggesting that alterations of GCase activity may be a common underlying feature of PD, similar to what has been proposed for changes in LRRK2 kinase activity.

Using a novel method of assessing GCase activity in dried blood spots, in 2015 Alcalay and colleagues reported a significant increase in GCase activity in carriers of the *LRRK2* G2019S mutation^14^, suggesting that carriers of the gain of function mutation have higher GCase activity and therefore the activities of the two enzymes are positively correlated in blood cells. Shortly after, using brain lysates from LRRK2 knock-out (KO) mice, we found that loss of LRRK2 results in decreased GCase levels, which corresponded to an increase in GCase-specific activity^15^. Because of the role played by LRRK2 in the vesicular and endo-lysosomal systems, several studies have followed to assess the link between mutant LRRK2 and GCase activities.

Recently, GCase activity of induced pluripotent stem cell (iPSC)-derived dopamine neurons from LRRK2-PD patients, carrying either the G2019S or the R1441C mutations, was found to be reduced compared to neurons from healthy controls, suggesting a negative correlation between these two activities^16^. There may be several possibilities for the discrepancy between this result and the results obtained from blood samples. First, there may be a cell-type-specific link between the activities of LRRK2 and GCase, manifesting in divergent ways in neuronal cells compared to peripheral blood immune cells. Second, methodological differences in the assessment of GCase activity (i.e., cell based vs. in vitro) may capture different aspects of this interplay. A further study on blood samples from PD-patients identified a correlation between the *LRRK2* variant M1646T and increased GCase activity^17^, consistent with the result we had obtained for *LRRK2* G2019S carriers^14^.

Interestingly, the interplay between LRRK2 and GCase was also identified in iPSC-derived astrocytes derived from PD patients carrying *GBA1* mutations, in which LRRK2 inhibition could rescue the lysosomal and inflammatory defects^18^.

To shed more light into the relationship between *LRRK2* and *GBA1*, here we assessed GCase activities in-lysate and in-cell across multiple LRRK2 cellular and in vivo models, including fibroblasts, plasma and peripheral blood mononuclear cell (PBMC) samples from idiopathic PD and *LRRK2* G2019S cohorts. Our findings support a model in which LRRK2 kinase activity is positively correlated with GCase activity but the mechanisms affecting lysosomal GCase are likely to be cell-type specific.

## Results

### G2019S LRRK2 mutation impacts GCase function in mouse brain

**Fig. 1:**
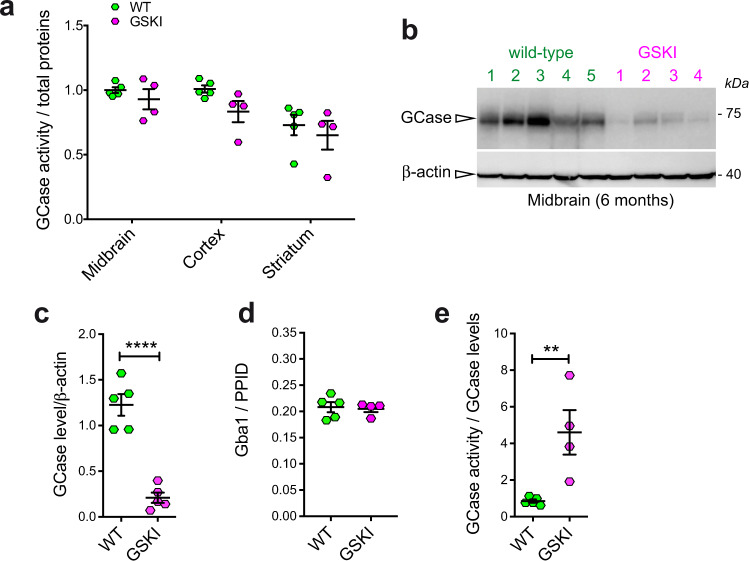
GCase activity and level measured in *LRRK2* G2019S KI mice brains. **a** GCase activity measured in brain extracts from different brain regions (midbrain, cortex and striatum) shows limited differences between WT and GSKI brains, while the difference is significant across the different measured tissue extract (*n* = 4–5 samples per genotype, two-way ANOVA test, *p* = 0.015, *F*(2, 21) = 9.013 for brain regions; *p* = 0.0708, *F*(1, 21) = 3.623 for genotype; data are expressed as mean ± SEM). **b** Western blot analysis of GCase protein levels and the relative β-actin loading control of tissue extracts from the midbrain for WT and KI mice. **c** Quantification of GCase protein level normalized to β-actin showed a significant decrease in GSKI midbrain compared with WT tissue extracts (*n* = 4–5 samples per genotype, Student *t* test, two-tailed ****p < 0.00001, Shapiro–Wilk normality test; data are expressed as mean ± SEM). **d** Quantification of GCase at the mRNA level via qPCR, using the housekeeping gene PPID (peptidylprolyl isomerase D), in GSKI midbrain compared with WT showed no significant difference between the two (*n* = 4–5 samples per genotype, two-way-ANOVA, *p* = 0.0353, *F*(2, 21) = 3.936 for genotype; *p* = 0.0580, *F*(1, 21) = 4.020 for mouse age; Tukey’s multiple comparison test indicates no differences between genotypes at the same age; data are expressed as mean ± SEM). **e** GCase activity was significantly higher in GSKI mice tissue when normalized to the GCase level (*n* = 4–5 samples per genotype, Student *t* test, two-tailed *p* = 0.0099, Shapiro–Wilk normality test; data are expressed as mean ± SEM).

To understand the effect of LRRK2 mutations on GCase function in brain tissues, we performed GCase activity assays and western blot analysis on brain lysates from different regions (midbrain, striatum, and cortex) in 6-month-old G2019S knockin (GSKI) and WT mice. GCase activity normalized by total proteins is different across brain regions, but not across genotypes (Fig. 1a, two-way ANOVA, Bonferroni post-test; brain region, *p* = 0.0015; genotype, *p* = 0.0708; interaction, *p* = 0.7089; *n* = 4–5). Specifically, the GCase activity measured in lysates from midbrain and cortex is higher compared to that observed in the striatum (Fig. 1a). Considering that dopaminergic neurons in the midbrain are the most vulnerable in PD, we focused on this region to further evaluate GCase levels. Strikingly, GCase protein levels are significantly lower in GSKI midbrain lysates as compared to WT (Fig. 1b, c).

To assess whether the differences in GCase protein level could be due to transcriptional differences between GSKI and WT mice, we performed qPCR analysis on midbrain samples and found that GCase was not affected at the mRNA level (Fig. 1d), supporting a post-translational mechanism underlying the reduced protein levels observed in GSKI midbrains (e.g. increased GCase degradation). Due to reduced GCase protein levels, specific GCase activity (i.e., activity normalized by the amount of enzyme) in GSKI midbrains was significantly higher than in WT midbrains (Fig. 1e).

### GCase activity is increased in affected LRRK2 G2019S mutation carriers

It has been previously reported that GCase activity is elevated in *LRRK2* G2019S carriers using a novel dried blood spot assay^14^. Here we tested if these results can be replicated in purified PBMCs from a similar cohort, hypothesizing that GCase measurements from PBMCs would be more accurate than measurements in dried blood spots. In this cohort, we previously found that in vitro LRRK2 kinase activity purified from PBMCs is significantly increased in both affected and healthy carriers of the *LRRK2* G2019S mutation compared to healthy controls and PBMCs from idiopathic PD (iPD) patients^19^.

**Fig. 2:**
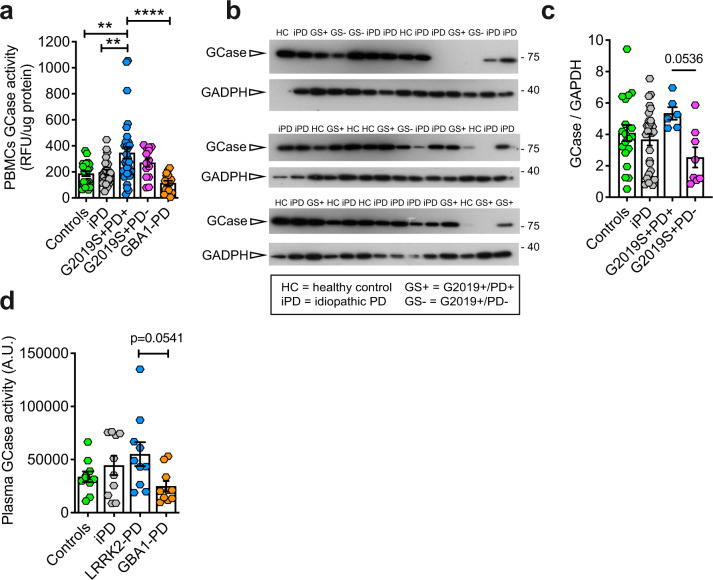
GCase activity and level measured in PBMCs and plasma from idiopathic PD patients, in *LRRK2* G2019S unaffected carriers and patients, in *GBA1* PD patients compared with controls. **a** GCase activity (normalized to total protein content) measured in PBMC extracts from the indicated PD cohort. While GCase activity in GBA1 mutation carriers is reduced, activity in affected G2019S-LRRK2 carriers is significantly elevated (one-way ANOVA, Tukey’s post-hoc tests; **p < 0.01, ****p < 0.0001; data are expressed as mean ± SEM). **b** GCase protein level as evaluated by western blot analysis in a subset of PBMCs for which the activity was evaluated (HC = healthy controls; iPD = idiopathic PD; GS+ = *LRRK2* G2019S carriers affected by PD; GS− = *LRRK2* G2019S carriers non-affected by PD; data are expressed as mean ± SEM). Note that samples showing absence of a GCase band were excluded from the analysis. **c** Quantification of GCase levels in PBMCs showing a non-significant increase in GS+ PBMCs compared to GS− (One-way ANOVA, Tukey’s multiple comparisons test, GS+ PD+ vs. GS+ PD− *p* = 0.0536; data are expressed as mean ± SEM). **d** GCase activity normalized by the total protein content measured in the plasma of idiopathic patients, in patients carrying *LRRK2* G2019S mutations or *GBA1* mutations. The trend is similar between plasma samples and PBMCs (10 individuals for each group; one-way ANOVA, Tukey’s multiple comparisons test; GS+ PD+ vs. GBA1-PD *p* = 0.0541; data are expressed as mean ± SEM).

As expected, GCase activity in PBMCs from carriers of *GBA1* mutations was lower, although not statistically significant, compared to PBMCs from healthy control subjects (Fig. 2a). This is likely due to the fact that PD patients carrying *GBA1* mutations still retain one copy of the functional gene that encodes the WT enzyme; moreover, some mutations associated to PD do not completely abolish GCase enzymatic activity. Likewise, as reported previously^20^, the GCase activity in PBMCs from iPD patients was not significantly different from healthy controls (Fig. 2a). Conversely, we found that GCase activity is increased in PBMCs from G2019S carriers, in agreement with our previous study^14^ (Fig. 2a). This was evident only in PBMCs from affected carriers of the *LRRK2* G2019S mutation, as non-manifesting carriers exhibit GCase activity similar to controls. We had insufficient amounts of PBMC extracts normally required for the evaluation of GCase protein level and for crude isolation of lysosomes for all the samples; thus, for these experiments, the fluorescent signal arising from GCase activity in the whole lysate was normalized to total protein expression. It is worth noting that the distribution of GCase activities in symptomatic G2019S carriers is larger compared to the other groups. This could be due to sampling variance, but could also suggest that when PD manifests, the G2019S mutation increases the likelihood that GCase activity is enhanced. Thus, in this mixed immune cell population, the link between LRRK2 kinase activity and GCase activity is not strictly tied to either LRRK2 mutation status, or disease status alone, but rather a synergistic effect between the two may be needed to alter the intrinsic activity of GCase. We excluded that the effect may be due differences in medications because the increase in the GCase activity would be observed also in iPD and *GBA1* PD patients, which instead does not occur.

For a subset of PBMCs, we were able to evaluate GCase protein level by western blot (Fig. 2b). The level of GCase observed in PBMCs does not change across the different genotypes (Fig. 2c), but LRRK2 G2019S PD patients exhibit a trend toward higher GCase protein level when compared to non-manifesting carriers (*p* = 0.0536, one-way ANOVA with Tukey’s post test), further suggesting that the interplay between *LRRK2* and GCase may change upon the manifestation of the disease. While this difference is not significant, the limited number of samples available could have hindered a stronger effect and this would warrant further investigations.

A similar trend was obtained by measuring GCase activity in plasma from a small pilot cohort (see the section “Materials and methods”) constituted by *LRRK2* G2019S PD patients, *GBA1* PD patients, iPD patients and controls (Fig. 2d) (one-way ANOVA with Tukey’s post test).

### Endolysosomal compartment is impaired in LRRK2 G2019S patient fibroblasts and GCase function is altered

**Fig. 3:**
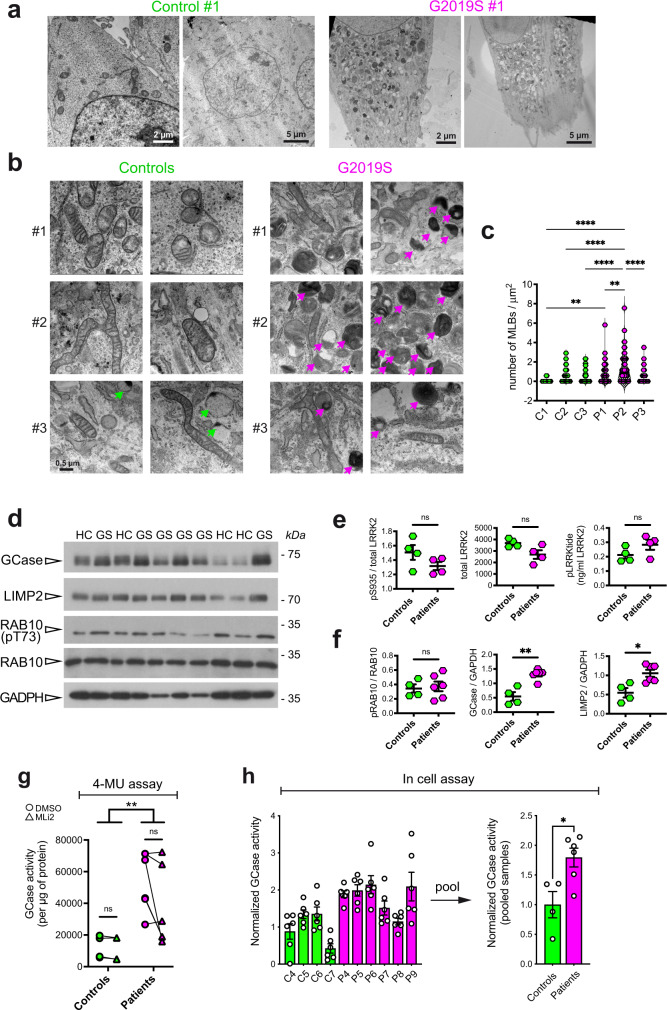
Ultrastructural analysis of endo-lysosomal compartment and GCase function in fibroblasts from *LRRK2* G2019S mutant compared with controls. **a** Low magnification (2–5 µm) TEM micrographs showing electron-dense structures accumulating in the *LRRK2* G2019S fibroblasts (Patient #1), while they are almost completely absent in control cells (Control #1). **b** High magnification (500 nm) TEM images three patient fibroblast cell lines and age and sex matched controls. Patient fibroblasts show accumulation of endo-lysosomal structures that resemble multilamellar bodies (MLBs). **c** Violin plots of the number of the MLBs per µm^2^ for each patient and each control cell line, showing that the number of MLBs is larger in *LRRK2* G2019S fibroblasts than in control cells (given a certain degree of variability across individuals) (one-way ANOVA, Tukey’s multiple comparisons test; **p < 0.01, ***p < 0.001, ****p < 0.0001). **d** Western blot analysis of GCase, LIMP2, pT731 RAB10 and total RAB10. **e** Quantification of ELISA assays for pS935 LRRK2, total LRRK2 (UDD3 antibody) and pLRRKtide (ng/ml LRRK2) (Mann–Whitney non-parametric test, *p* > 0.05; data are expressed as mean ± SEM). **f** Quantification of western blots in **e** (Mann-Whitney non-parametric test, controls vs. patients: pRAB10 *p* > 0.999, GCase *p* = 0.0095, LIMP2 *p* = 0.019; data are expressed as mean ± SEM). **g** CGase activity assessed with the in-lysate 4-MU method. G2019s patients display increased activity compared to controls while MLi-2 treatment has no effect in both genotypes (two-way ANOVA, genotype effect *p* = 0.001 *F*(1, 16) = 16.04, treatment effect *p* = 0.2891 *F*(1, 16) = 1.202). **h** GCase activity assessed with the in-cell method from Benz 2021 protocol is significantly increased in G2019S patients (Mann–Whitney non-parametric test, *p* = 0.0381; data are expressed as mean ± SEM). Each dot represents one subject and the value is the average of two biological replicates each with three technical replicates.

To further investigate the role of the hyperactive *LRRK2* G2019S mutant on GCase behavior in another type of patient-derived cells, we analyzed a series of three PD-patient fibroblasts carrying G2019S LRRK2 mutation compared to three age-matched, healthy controls. Patient cell lines were first analyzed by transmission electron microscopy (TEM) in comparison with matched controls, to identify macroscopic alterations in the endo-lysosomal compartment in relationship to LRRK2 mutation. Representative TEM micrographs reported in Fig. 3a, b show a striking accumulation of electron dense or lamellar structures in LRRK2 patient cells. They are likely endo-lysosomes engulfed with undigested materials, autophagic vacuoles and multilamellar bodies (Fig. 3a, b), which are all typical hallmarks of altered autophagic lysosomal pathways. Interestingly, multilamellar bodies (MLBs) were also found in fibroblasts from PD-patients carrying the N370S mutation in the GBA gene^21,22^. Quantification of the number of MLBs per area across the different cell lines revealed a significant increase of these structures in patient lines compared to controls, with G2019S carrier lines showing a higher degree of variability as compared to controls (Fig. 3c).

To further investigate the relationship between GCase function and LRRK2 kinase activity we used larger series of fibroblast lines, consisting of 4 control lines from healthy subjects and 6 LRRK2 G2019S fibroblasts from PD patients, which allowed us to better characterize the relationship between LRRK2 and GCase in the fibroblast model.

The level of GCase and LIMP2 and the phosphorylation of RAB10 were evaluated by western blot in these new set of fibroblasts (Fig. 3d). LRRK2 levels, phosphorylation of S935 and activity (phospho-LRRKtide) were further investigated using ELISA assays, which show a non-significant increase in activity and decrease in pS935, as predicted (Fig. 3e). Strikingly, quantification of western blots revealed that both GCase and LIMP2 levels are significantly higher in patients compared to control fibroblasts (Fig. 3f). Interestingly, we were unable to detect significant changes in LRRK2 kinase activity when looking at RAB10 phosphorylation at T73, in line with a recent study also showing that peripheral blood neutrophils carrying G2019S LRRK2 fail to exhibit increased phosphorylation of RAB10^23^.

We next evaluated GCase activity using the classical assay with the 4-MU substrate and found a significant increase in GCase enzymatic function in lysates from G2019S patient fibroblasts (Fig. 3g; two-way ANOVA, genotype *p* = 0.001). Also in this series, LRRK2 G2019S fibroblasts exhibit a large variability, which may be explained by putative differences in disease manifestations similarly to what we observed in PBMCs from G2019S carriers or PD patients. In both control and patient cells, the treatment with the LRRK2 inhibitor MLi-2 at 100 nM for 90’ did not show a significant effect on the GCase activity (Fig. 3g and Supplementary Fig. 1).

To evaluate GCase activity also in the cellular context, we employed a method following the protocol by Benz et al. (2021)^24^ based on the use of the GCase fluorescence substrate PFB-FDGlu. The GCase substrate was given to the cells 6 h before the assay, it specifically accumulates in lysosomes and it becomes fluorescence proportionally to the GCase activity in the organelles. By lysing the cells before recording the fluorescence, this method eliminates the possible differences in lysosomal pH between controls and G2019S mutation carriers^25^. As reported in Fig. 3h, there is a clear trend toward an increase in GCase activity in *LRRK2* G2019S fibroblasts compared to controls, confirming the results of the in-lysate assay. When the average value of individual controls and patients are pooled, there is a significant (about 75%) increase in the in-cell GCase activity in LRRK2 G2019S fibroblasts compared to control cells (Mann–Whitney test, *p* = 0.0381) (Fig. 3h). Taken together, these results indicate that fibroblasts from LRRK2 G2019S patients possess increase GCase enzymatic activity.

### GCase activity is increased in iPSC-derived dopaminergic neurons from G2019S LRRK2 patients

**Fig. 4:**
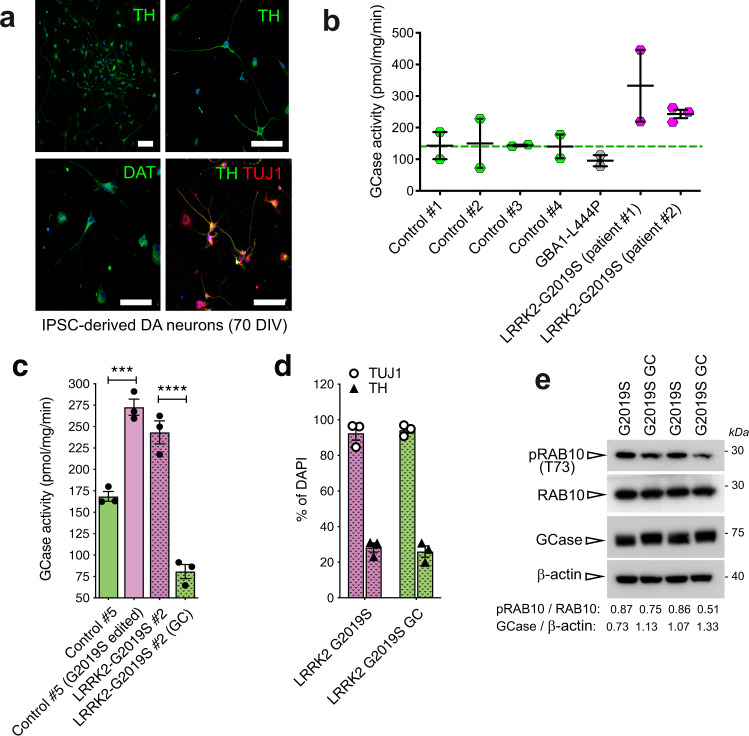
iPSc-derived neurons from *LRRK2* G2019S PD patients and *GBA1* PD patient show altered GCase activity compared to neurons derived from healthy subjects. **a** Representative confocal images of iPSC-derived neurons stained with TH, DAT, and TUJ1 antibodies to verify proper differentiation to dopaminergic cells. **b** GCase activity measured in iPSC-derived neurons obtained from four healthy controls, one *GBA1* L444P PD patients and two *LRRK2* G2019S PD patients (2–3 replicates per individual). Data show that *GBA1* PD patients have a reduced GCase activity compared with controls, while *LRRK2* PD patients present increased GCase activity (data are expressed as mean ± SEM). **c** GCase activity measured in iPSC-derived dopaminergic neurons obtained from one healthy control in which the *LRRK2* G2019S was introduced (Control #5 G2019S edited) show an increased enzymatic activity compared with the non-edited neuronal cells (Control #5). Similarly, GCase activity was reduced to a level similar to the Control #5 iPSC-derived neurons in LRRK2 G2019S dopaminergic cells (LRRK2-G2019S #2) upon gene correction (LRRK2-G2019S #2 GC). One-way ANOVA with Tukey’s post-test (**p < 0.01; ****p < 0.0001; data are expressed as mean ± SEM). **d** Efficiency of the differentiation into TH- positive neurons in LRRK2-G2019S #2 and LRRK2-G2019S #2 GC is about 30% of neurons (data are expressed as mean ± SEM). **e** Western blot showing pRAB10 and GCase levels in LRRK2-G2019S #2 and LRRK2-G2019S #2 GC. GCase level and pRAB10 were expressed as ratio of total RAB10 and β-actin, respectively.

We next evaluated the role of LRRK2 kinase activity on GCase function in a human-derived disease-relevant model. To this aim, we took advantage of iPSC-derived dopaminergic neurons. iPSC lines of PD patients and controls stained positive for stem cell markers (SOX2, OCT4, TRA-1–60, SSEA4). Karyotype analysis excluded genetic abnormalities of reprogrammed iPSCs. Dopaminergic neurons differentiated from all iPSC lines cultured up to 70 days in vitro-stained positive for neuronal (TUJ1) and dopaminergic (TH, DAT) markers, confirming appropriate differentiation (Fig. 4a). The efficiency of dopaminergic neuron differentiation with this protocol was described in ref. ^26^: >55% of TUJ1-positive cells are also positive for the catecholaminergic marker TH and no differences in DA neuron yields across lines and batches of differentiation was observed.

As a first screen, we measured GCase activity in iPSC-derived neurons carrying mutations in different PD genes, i.e., *GBA1* (L444P), *LRRK2* (G2019S) and compared to five different healthy controls. In line with results obtained in PBMCs, and as expected, iPSC-derived dopaminergic neurons carrying L444P mutation in *GBA1* gene showed decreased activity of GCase, compared to healthy controls (Fig. 4b). Consistent with data on GCase activity in PBMCs from PD patients carrying G2019S *LRRK2* mutation, two lines of iPSC-derived dopaminergic neurons carrying the G2019S mutation in *LRRK2* gene showed increased GCase activity (Fig. 4b). To demonstrate that the effect on GCase activity is genuinely mediated by the *LRRK2* G2019S mutation, dopaminergic neurons were differentiated from an isogenic control line with wild type LRRK2 (LRRK2 #2 GC) corrected from iPSCs of a *LRRK2* G2019S patient (LRRK2 #2) and from an isogenic line from a control subject (CTR5) edited to carry *LRRK2* G2019S mutation (CTR5 G2019S). The increase in the GCase activity observed in the LRRK2 mutated line was significantly lowered in the isogenic control with corrected LRRK2. Similarly, the introduction of the *LRRK2* G2019S mutation in the control line resulted in an increased GCase activity (Fig. 4c). The efficiency of the differentiation protocol for this new cell lines was evaluated separately and about 30% of neurons were positive for the TH marker (Fig. 4d). As evaluated by western blot, G2019S LRRK2 iPSC-derived neurons show a trend toward an increase in pRab10 at T73 and a parallel decrease in GCase level, compared to the relative gene corrected neurons (Fig. 4e). Taken together, we collected multiple lines of evidence that GCase activity is increased in different cell types, including iPSCs-derived dopaminergic neurons and peripheral immune cells, expressing hyperactive G2019S LRRK2.

### Genetic or pharmacological inhibition of G2019S LRRK2 kinase activity in HEK293T cells leads to a decrease in GCase activity

**Fig. 5:**
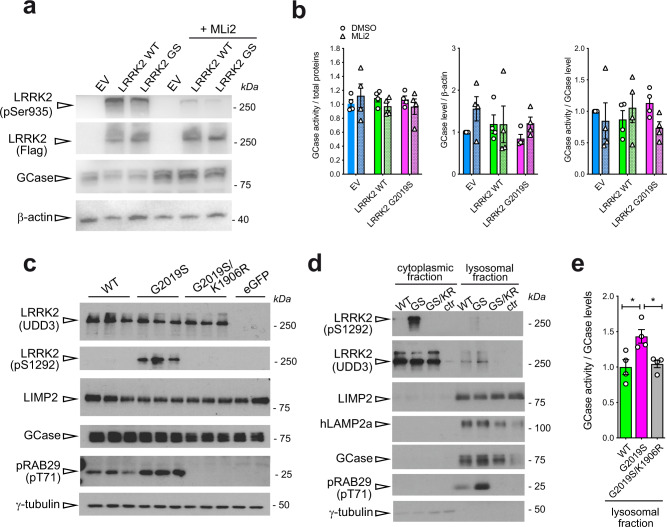
GCase activity and level is altered in HEK293T cells in which LRRK2 kinase activity is pharmacologically or genetically manipulated. **a** Representative western blot of LRRK2-Flag, pSer935 LRRK2 and GCase in HEK293 cells overexpressing empty vector (EV), LRRK2 WT and LRRK2 G2019S and treated with the LRRK2 inhibitor MLi2 100 nM for 90’. **b** From left to right, quantification of the GCase activity (normalized by total proteins), GCase levels normalized by β-actin and GCase activity normalized by GCase level from western blot. No significant differences across tratments and genotypes (Two-way ANOVA; data are expressed as mean ± SEM). **c** Representative western blot of HEK293T cells over-expressing WT, G2019S, or G2019S/K1906R (kinase-dead) LRRK2 probed for total (UDD3) and phosphorylated (pS1292-LRRK2) LRRK2, LIMP1, GCase, phosphorylated Rab29 (T71), and γ-tubulin. LRRK2 kinase function, increased autophosphrylation and phosphorylation of endogenous Rab29 in cells expressing G2019S-LRRK2 is prevented by the kinase inactivating mutation (K1906R). **d** Representative western blot of cytoplasmic and lysosomal fraction of HEK293T cells over-expressing WT, G2019S, or G2019S/K1906R (kinase-dead) LRRK2 probed for total (UDD3) and phosphorylated (pS1292-LRRK2) LRRK2, LIMP1, GCase, phosphorylated Rab29 (T71), and γ-tubulin. **e** Quantification of GCase activity normalized by GCase level in the lysosomal fraction of HEK293T cells over-expressing WT, G2019S, or G2019S/K1906R (kinase-dead) LRRK2 showed a significant increase in G20129S samples compared with LRRK2 WT or with the kinase-dead mutant. (One-way ANOVA with Tukey’s post-test; *p < 0.05; data are expressed as mean ± SEM).

We next examined if the effect of the LRRK2 G2019S mutation on GCase activity specifically depends upon the increased kinase activity associated with this mutation. To this aim, we employed HEK293T cells transiently over-expressing LRRK2 WT or G2019S compared to control cells and evaluated GCase activity and level upon pharmacological inhibition of LRRK2 kinase activity using MLi-2 (Fig. 5a, b). As shown in Fig. 5b, overexpression of LRRK2 WT or G2019S does not affect GCase activity when normalized to total proteins or total GCase levels. Moreover, 100 nM MLi-2 treatment for 90’ did not significantly affect GCase activity or levels, although there was a non-significant decrease of GCase activity upon MLi-2 treatment in LRRK2 G2019S expressing cells (Fig. 5b). The effect of MLi-2 inhibition on LRRK2 was confirmed by the reduction of LRRK2 phosphorylation at Ser935 (Fig. 5a).

As these measurements are performed in the total cell lysate, we next focused on the lysosomal fraction. To this aim, we transiently overexpressed LRRK2 WT, mutant LRRK2 G2019S, or a LRRK2 kinase-dead double mutant G2019S/K1906R, in HEK293T cells and assessed GCase activity in crude lysosomal fractions. To confirm that the over-expressed G2019S and G2019S/K1906R mutants elicited the expected effects on LRRK2 kinase activity, we assessed autophosphorylation of LRRK2 at Ser1292. Cells expressing LRRK2 G2019S exhibit increased autophosphorylation at Ser1292, as expected, which is reversed by the kinase-dead form of this mutant (Fig. 5c). In crude lysosomal fractions, we could detect total LRRK2 (Fig. 5d), as well as a faint band corresponding to pS1292-LRRK2 in lysosomes of cells expressing *LRRK2* G2019S, and increased pT71-RAB29. The lysosomal localization of both phosphorylated as well as total LRRK2 was dependent on its kinase activity, as the K1906R-inactivating mutation blocked these accumulations (Fig. 5d). Importantly, we found a significant increase in GCase activity normalized to GCase level in enriched lysosomal fractions of cells expressing LRRK2 G2019S (Fig. 5e). The kinase-dependency of this induction was confirmed by the reversal of this phenotype in cells expressing kinase-dead G2019S/K1906R LRRK2. Taken together, these data indicate that LRRK2 G2019S increases lysosomal GCase activity.

### Lysosomal GCase activity in LRRK2 knockout macrophages

**Fig. 6:**
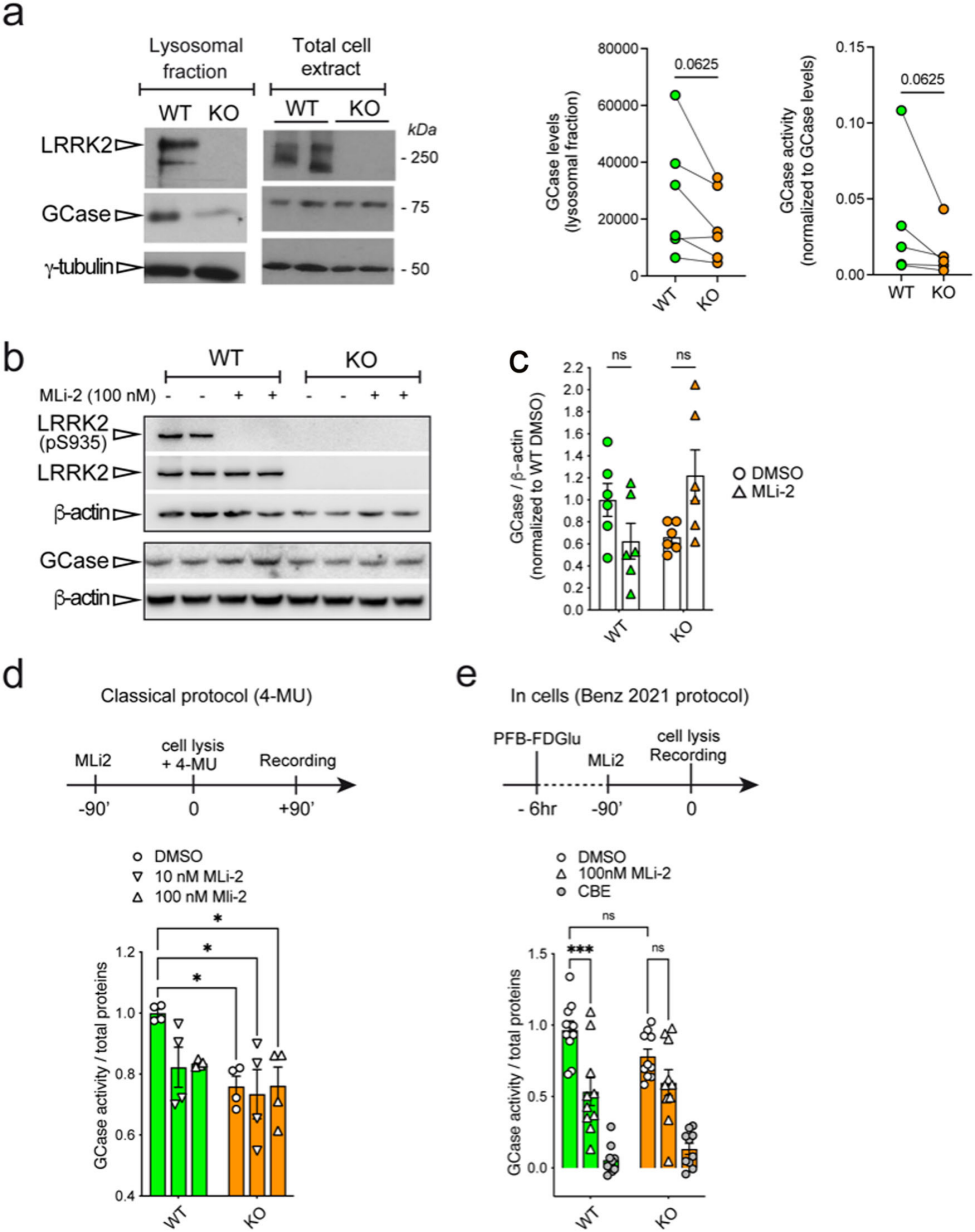
GCase activity and level is reduced in lysosomal extracts from LRRK2 KO RAW264.7 cells. **a** RAW264.7 cells, WT and LRRK2-deficient (KO) were processed for western immunoblotting of the total cell extract and crude lysosomal fraction, and the membranes probed for LRRK2, GCase, and γ-tubulin. GCase levels are unchanged in the cell lysates of LRRK2 KO cells, compared to WT cells; however, they are reduced (though non-significantly) in crude lysosomal fractions from KO RAW264.7 cells. On the right, quantification of GCase levels (*n* = 6 independent replicates, mean ± SEM: WT = 0.03441 ± 0.01905, KO = 0.01460 ± 0.007323; *p* = 0.0625, Wilcoxon matched-pairs signed rank test, effectiveness of pairing *p* = 0.0417) and activity normalized by GCase levels (WT = 28121 ± 8744, KO = 17777 ± 5171; *p* = 0.0625, Wilcoxon matched-pairs signed rank test, effectiveness of pairing *p* = 0.0083; mean ± SEM) in the lysosomal fraction. **b** Western blot analysis of LRRK2 WT vs. KO RAW total lysates of pS935 LRRK2, total LRRK2 (MJFF3 antibody), GCase and β-actin loading control. Treatment with 100 nM MLi-2 for 90’ results in complete dephosphorylation of S935 as expected (*n* = 3 biological replicates each with 2 technical replicates; two-way ANOVA, Tukey’s multiple comparisons test, WT DMSO vs. WT MLi-2 *p* = 0.3799; KO DMSO vs. KO MLi-2 *p* = 0.0995; genotype effect *p* = 0.4325 *F*(1, 20) = 0.6417, treatment effect *p* = 0.5732 *F*(1, 20) = 0.3280). **c** Quantification of GCase levels does not show differences across genotypes and treatments (two-way ANOVA, Tukey’s multiple comparison’s test; *p* > 0.05; data are expressed as mean ± SEM). **d** GCase activity assessed with the in-lysate 4-MU protocol comparing WT vs. KO RAW cell lysates treated for 90’ with DMSO, 10 or 100 mM MLi-2 (*n* = 2 biological replicates each with 2 technical replicates, two-way ANOVA, Tukey’s multiple comparison’s test; WT:DMSO vs. KO:DMSO *p* = 0.0388; WT:DMSO vs. KO:10 nM MLi-2 *p* = 0.0191; WT:DMSO vs. KO:100 nM MLi-2 *p* = 0.0413; WT:DMSO vs. WT:100 nM MLi-2 *p* = 0.25; genotype effect *p* = 0.0051 *F*(1, 18) = 10.15, treatment effect *p* = 0.1416 *F*(2, 18) = 2.184; data are expressed as mean ± SEM). **e** GCase activity assessed with the in-cell protocol from Benz et al. (2021) comparing WT vs. KO RAW cell lysates treated for 90’ with DMSO, 100 nM or the GCase inhibitor CBE (*n* = 3 biological replicates with 3–4 technical replicates, two-way ANOVA, Tukey’s multiple comparison’s test; WT:DMSO vs. WT: 100 nM MLi-2 *p* = 0.0004; KO:DMSO vs. KO:100 nM MLi-2 *p* = 0.3883; WT:DMSO vs. KO:DMSO *p* = 0.3901; other non-informative comparisons, both *p* > 0.05 and <0.05, were omitted; genotype effect *p* = 0.7918 *F*(1, 54) = 0.07040; treatment effect *p* < 0.0001 *F*(2, 54) = 8.28; data are expressed as mean ± SEM).

To further complement and expand these findings, we took advantage of the macrophage cell model RAW264.7 where LRRK2 biology is well-characterized^27^. One advantage of this model is that macrophages express both GCase and LRRK2 at high levels, allowing to study the impact of LRRK2 kinase activity on GCase function without over-expressing LRRK2. Moreover, in this cell type, LRRK2 exerts crucial functions in the regulation of immune and inflammatory responses^27^, but also in the control of lysosomal damage^28,29^. Based on the previous experiments suggesting an effect in the lysosomal compartment, we assessed GCase level and activity using the 4-MU substrate-based assay in crude lysosomal preparations isolated from LRRK2 WT and knockout (KO) macrophages. GCase levels are reduced by about 50% in crude lysosomal preparation from LRRK2 KO cells (Fig. 6a; WT = 0.03441 ± 0.01905, KO = 0.01460 ± 0.007323; *p* = 0.0625, Wilcoxon matched-pairs signed rank test, effectiveness of pairing *p* = 0.0417), while in total extract the trend is similar but not significant (Fig. 6b). Interestingly, when normalized to GCase level, the activity of the enzyme in LRRK2 KO cells compared to WT is lower in the lysosomal fraction, even though not statistically significant (Fig. 6a; WT = 28121 ± 8744, KO = 17777 ± 5171; *p* = 0.0625, Wilcoxon matched-pairs signed rank test, effectiveness of pairing *p* = 0.0083). This may suggest that by knocking out LRRK2, GCase localization at the lysosome is impaired, either by altering its trafficking or its degradation. To gain a broader picture of the impact of LRRK2 KO on GCase activity, we compared the in vitro and in-cells protocols to assess GCase enzymatic activity. MLi-2 was used to understand the contribution of LRRK2 kinase activity.

When using the classical in-lysate 4-MU-based assay, we found that the GCase activity is significantly reduced in LRRK2 KO cells compared to WT to a similar extent to the measured activity when LRRK2 WT cells are treated with 100 nM MLi-2 (Fig. 6d; two-way ANOVA, Tukey’s multiple comparison’s test; WT:DMSO vs. KO:DMSO *p* = 0.0388; WT:DMSO vs. KO:10 nM MLi-2 *p* = 0.0191; WT:DMSO vs. KO:100 nM MLi-2 *p* = 0.0413; WT:DMSO vs. WT:100 nM MLi-2 *p* = 0.25). No impact of MLi-2 treatment is observed in LRRK2 KO cells, as expected.

When exploiting the in-cells protocol previously used in fibroblasts (Fig. 3e)^24^, we found a non-significant decrease in GCase in KO cells, but when treating LRRK2 WT cells with 100 nM Mli-2 there was a significant decrease in the GCase activity in the lysosomes of LRRK2 WT cells (Fig. 6e), further corroborating the previous results (Fig. 6a). The same does not occur for the LRRK2 KO cells (two-way ANOVA, Tukey’s multiple comparison’s test; WT:DMSO vs. WT: 100 nM MLi-2 *p* = 0.0004; KO:DMSO vs. KO:100 nM MLi-2 *p* = 0.3883; WT:DMSO vs. KO:DMSO *p* = 0.3901; other non-informative comparisons, both *p* > 0.05 and <0.05, were omitted).

## Discussion

In this work we showed that an overall positive correlation exists between LRRK2 kinase activity and GCase hydrolytic activity ex-vivo using a wide range of PD-models, as well as human clinical samples and patient-derived iPSC dopaminergic neurons. Our assessments reflect the intrinsic activity of GCase outside the cellular context upon normalization by GCase protein levels (when possible) or in-cell GCase activity measured on the enzyme resident in the lysosomes. Overall, increment of LRRK2 kinase activity due to G2019S mutation positively correlates with increases in GCase enzymatic activity, while the level of the enzyme is differently affected depending on the model explored. In particular, there seems to be a marked difference between mouse brain tissue or patient iPSC-derived neurons carrying the G2019S mutation, in which GCase level is reduced, and other PD-derived cell models (PBMCs and fibroblasts), in which the protein level is increased or unchanged, suggesting that LRRK2 G2019S affects GCase steady state levels differently in brain versus peripheral tissues.

Interestingly, GCase protein level is reduced also in the lysosomal fraction of extracts of LRRK2 KO macrophages and a similar trend is present also in the total cell lysates. These results are in agreement with our previous study where we detected reduced GCase protein levels in the whole brain of 12-month-old LRRK2 KO mice and increased GCase activity when normalized by GCase levels^15^. Recently, the group of Morari and colleagues failed to detect a difference in striatal GCase expression levels in 3- or 12-month-old WT and KO mice^30^; however, striatal GCase activity was reported to be increased in KO and kinase dead KI mice. In contrast, G2019S-KI mice did not exhibit differences in striatal GCase activity. The difference in findings among this report, our earlier study in vivo, and the current study are likely due to methodological differences, such as the normalization of GCase activity and the use of a specific GCase inhibitor (e.g. CBE) to reveal changes in specific activity.

One possible interpretation for the reduction of the GCase protein localized at the lysosomes is that the amount of GCase that is correctly trafficked in RAW264.7 LRRK2 KO cells is reduced, while the overall GCase produced in cells remain unchanged. Another possibility is that the increased protein degradation generally observed in LRRK2 KO^31^ affects the amount of GCase in the lysosomes. However, since we observed that GCase activity (4-MU assay) is also reduced in LRRK2 KO cells, we predict that GCase protein may be partially unfolded or it may lack posttranslational modifications such as glycosylation, that are key for both the trafficking and the activity of the protein^32^. This is further corroborated by the fact that in GSKI mice midbrains we observed a reduced GCase protein level, but no changes at the mRNA level, as evaluated by qPCR analysis. Thus, LRRK2 regulation of GCase is likely to be linked to the trafficking or the degradation of the lysosomal enzyme.

Consistently with the 4-MU in lysate activity assay, by inhibiting LRRK2 kinase activity with 100 nM MLi-2 we observe a reduction in the GCase activity of LRRK2 WT RAW264.7 cells also when measuring in-cells GCase activity, which further correlate the two proteins at the lysosome.

Further supporting that alterations in GCase are correlated to LRRK2 kinase activity, the lysosomal-enriched fraction of HEK293T cells expressing the G2019S LRRK2 exhibited increased GCase activity in cells that was rescued by genetic introduction of a kinase dead point mutation (G2019S/K1906R). Consistently, these experiments support the positive correlation between GCase hydrolytic activity and LRRK2 kinase activity in the lysosome. Interestingly, it seems that the increase of the intrinsic GCase activity is present only when the G2019S LRRK2 protein is overexpressed, as it does not occur with the WT, suggesting a specific or experimentally detectable effect of the hyperactive mutant, which is rescued by both genetic and pharmacological inhibition of the kinase activity.

In addition to these cellular models, we assessed GCase activity in patient-derived models more relevant for LRRK2-PD pathology: PBMCs, plasma, fibroblasts and iPSC-derived neurons. Overall, a positive correlation between LRRK2 kinase and GCase hydrolytic activities is observed in all these systems, although to different extents. In PBMCs, *LRRK2* G2019S PD patients display an increased GCase activity compared with healthy controls^15^, while the activity of GCase in PBMCs from non-manifesting *LRRK2* G2019S carriers is similar to that of controls. This may suggest that a synergistic effect of the mutation and of the disease status occurs, at least in this immune cell population. Another possible explanation is that GCase activity alterations in subjects carrying *LRRK2* G2019S mutations are measurable in this cell type only when the disease is already clinically manifested, suggesting that GCase activity in these patients may be used as a marker for disease progression or manifestation. We excluded a contribution from medications to this increase, as it should be present also in iPD and in GBA1-PD patients. Of interest, phosphorylation of RAB10 is not increased in PBMCs from G2019S patients, in agreement with recent findings in patient peripheral neutrophils^23^ and lung and kidney from Lrrk2 G2019S knockin mice^33^. Similar to PBMCs, a comparable trend is observed in plasma, where manifesting *LRRK2* G2019S carriers exhibit higher (although not significant) GCase activity as compared with iPD or healthy controls. It has been previously reported that lysosomal enzyme activity can be measured in the plasma of patients^34^, suggesting that these proteins are likely to be secreted in certain conditions. In particular, this may occur via lysosomal exocytosis, which is a Ca^2+^-regulated process important for plasma membrane repair and secretion^35^. This may be relevant and deserves further investigation to understand if LRRK2 is involved in the regulation of the pathway and to evaluate if GCase measurement in the plasma (together or alternatively to PBMCs) could be used as a biomarker at least for certain PD forms and may allow patient stratification. Given the participation of LRRK2 in such pathways, it is reasonable to envision a role for LRRK2 in regulating GCase release or secretion, which would support a possible general impact of LRRK2 on GCase trafficking.

GCase activity is significantly reduced in *LRRK2* G2019S gene-corrected iPSC-derived neurons, whereas it is increased in control iPSC-derived neurons in which the G2019S mutation has been introduced. This suggests that, at least in this cell type, increased LRRK2 kinase activity is itself sufficient to impact on GCase and may be a peculiar feature of dopaminergic neurons, associated with their prominent vulnerability to cell death in PD.

In contrast to our current and previous findings showing no difference in activity in mixed PBMCs from two independent iPD cohorts^20^, the group of Dzamko reports reduced GCase activity in isolated CD14+ monocytes from idiopathic PD patients^11^. A major difference from the two studies is that Atashrazm and co-authors used an in-cell (PFB-FDGlu) recording method for assessing GCase activity, but also raises the possibility that cell-type-specific alterations in GCase activity may exist in the different immune cell sub-types, since PBMCs are a more heterogeneous cell population than CD14+ monocytes. Further studies assessing GCase activity (using both methodologies) in purified monocytes from LRRK2 mutation carriers, are warranted. Our finding of decreased GCase activity in PD-patients carrying *GBA1* mutations is consistent with previous reports in other models^9,36,37^. One possibility is that in both G2019S LRRK2 and GBA1-PD patients is more the misfolding and impaired trafficking that contribute to the PD pathogenesis, likely inducing ER stress, as recently shown by Stojkovska et al. (2022) in PD-midbrain neurons presenting α-synuclein aggregation and associated GCase-impaired proteostasis^38^.

It has been reported that deficiency in GCase activity leads to the accumulation of α-synuclein^39^. The increased GCase activity in *LRRK2* G2019S patients may explain, at least in part, the lack of Lewy Bodies in a subpopulation of LRRK2-patients^40^, a hypothesis that requires further investigations. In the case of *LRRK2* G2019S PBMCs and iPSC-derived neurons, normalization by GCase expression levels could not be performed. However, if the trend in GCase protein levels is consistent with the other models, GCase intrinsic activity may further increase also in these systems.

In human fibroblasts, as for all other human-derived cell types, the interindividual variability was even more pronounced. By analyzing a larger number of controls and patients, we were able to identify an increase in GCase activity in mutant LRRK2-patient derived fibroblasts, further supporting the idea that in certain cell types as iPSC-derived dopaminergic neurons the effect is more pronounced than in others. What is striking is instead the presence of MLBs in the *LRRK2* G2019S cells that we could analyze by TEM that are almost completely absent in control fibroblasts. This resembles the behavior of fibroblasts from PD patients carrying the N370S *GBA1* mutation, which have reduced GCase activity and accumulate MLBs^21^. This may indicate that LRRK2 mutant-expressing cells fail to mount an adequate response via upregulation of GCase activity transition to a state of dysfunction of the ALP.

Technical differences in the methods used to measure GCase activity and to normalize it may account for the different results reported by Krainc, Morari and Dzamko groups^11,16,30^. In those studies, lysosomal GCase activity was measured in iPSC-derived *LRRK2* G2019S neurons using the live-cell probe PFB-FDGlu rather than in-lysate GCase activity, which is more akin to assess the intrinsic activity of the isolated enzyme, possibly reflecting certain post-translational modifications of the protein itself. In contrast, a cellular-based GCase probe would not just reflect the net effect of the intrinsic GCase activity, but also the activities of other lysosomal enzymes and co-factors, the trafficking of GCase to the lysosome, and lysosomal pH. It should also be noted that the cellular GCase probe utilized in these studies is also sensitive to lysosomal pH, which is known to be altered in the context of *LRRK2* G2019S expression^25,41^. Additionally, the cellular uptake of this probe, through pinocytosis, may also be affected by LRRK2 activity^42^, further complicating the interpretation. In the present study, we also measured in-cells GCase activity using the PFB-FDGlu live-cell probe but we followed a novel method recently published by Benz et al. This method is predicted to overcome the problem of the pH-dependency, since cells are lysed at the end of the treatment but before the measurement (which then occurs at the lysis buffer pH) and to minimize the risk of altered substrate uptake by incubating the cells for longer time with PFB-FDGlu before the measurement (6 h versus 60 min).

The mechanisms by which LRRK2 kinase activity mediates GCase function seems to be related to both GCase level and its intrinsic activity. Given its prominent role in membrane trafficking, it is tempting to speculate that one potential mechanism for the effects of LRRK2 activity on GCase function is due to altered trafficking to the lysosome. Accordingly, in our enriched lysosomal preps, we detect a significant increase in GCase activity in vitro in the presence of *LRRK2* G2019S. To determine whether the impact of LRRK2 on lysosomal GCase content and activity is specific, it will be critical to evaluate whether GCase transporters or co-factors, such as LIMP2 and the M6P receptor, or other lysosomal proteins are affected in LRRK2 models. Other possibilities, as previously mentioned, include an impact of LRRK2 kinase activity on GCase glycosylation, which is largely unexplored, or on degradative processes, as for example by impacting on the E3 ubiquitin ligase thyroid hormone receptor interacting protein 12 (TRIP12) that was recently found to be crucial in controlling GCase proteasome degradation^43^. Further analysis will be needed to define the molecular mechanisms that determines the outcome of our observations.

Finally, carriers of both *GBA1* and *LRRK2* mutations are frequently reported, particularly within specific ethnic groups such as the Ashkenazi–Jewish community. While clinical reports in the literature of such double carriers are few, in one study it was found that the G2019S mutation in mutant *GBA1* carriers appeared to reduce the likelihood of patients developing non motor symptoms^44^. In another recent study, *LRRK2* G2019S/*GBA1* PD patients showed a more benign course of the disease with respect to both motor and non-motor symptoms when compared to *GBA1*-PD patients^45^.

Thus, our findings are not in disagreement with the clinical evidence and may suggest that a positive correlation between LRRK2 and GCase activity may account for the milder (or not cumulative) phenotype observed in carriers of both LRRK2 and GBA1 mutations^44,46^. However, this will need to be experimentally proven.

Clearly, more studies are warranted to uncover the link between these two key players in PD pathogenesis and progression and the precise molecular mechanisms by which this interplay occurs in the different idiopathic and genetic forms of the disease, and in different cell types. Indeed, LRRK2 kinase activity is a positive regulator of different inflammatory pathways and it has been shown to be protective in some types of infections, while detrimental in PD-related contexts. Thus, the direction of LRRK2-GCase interplay may not be generalizable and LRRK2-based therapies in PD should take into account the different effects of brain vs. peripheral LRRK2 activity.

## Methods

### Patient demographics

The PBMCs and plasma samples assessed in this study were collected from patients and control subjects at the Movement Disorder division in the Department of Neurology at Columbia University Irving Medical Center. The demographic and clinical data of the participants have been described in more detail elsewhere^19^. Participants were screened for the *LRRK2* G2019S mutation, as well as several *GBA1* mutations and variants. All samples were collected under informed consent: participants provided written informed consent to take part in the study and the study protocol was approved by the IRBs of both Columbia University (CUIMC) as well as the Biomedical Research Foundation of the Academy of Athens (BRFAA).

### PBMC isolation

PBMCs were isolated using standard protocols. Briefly, sodium citrate Vacutainer tubes (BD) were used to collect whole blood from study participants. Blood was diluted 1× in sterile PBS and transferred to Ficoll-containing Leucosep tubes (Griener) and centrifuged at 1000 × *g* for 10 min at room temperature. The upper plasma phase was then extracted and centrifuged at 300 × *g* for 15 min at room temperature. The banded cells were collected and washed 1× in PBS and centrifuged again at 300 × *g* for 10 min before counting and aliquoting, in RPMI with 40% FBS and 10% DMSO, at 3 × 10^6^ viable cells per cryovial. Frozen cells were stored at −80 °C. In total, we report results from 70 participants, including 20 idiopathic PD, 18 healthy controls, 10 GBA1-PD, 4 non-manifesting GBA1, 8 LRRK2-PD, 9 non-manifesting LRRK2, and one subject with PD and both GBA1 and LRRK2.

### Plasma isolation

Whole blood was collected in EDTA vacutainer tubes, which were centrifuged at 1500 × *g* for 15 min at 4 °C, from which 1 mL plasma aliquots were extracted within 60 min of collection. Samples were stored in a −80 °C freezer until processing. In total, we report results from 40 subjects, including 10 idiopathic PD, 10 healthy controls, 10 GBA1-PD, and 10 LRRK2-PD subjects.

### Human-derived fibroblasts

The first set of cell lines (Fig. 3a–d) were obtained at the “Cell line and DNA biobank from patients affected by genetic diseases” (Istituto G. Gaslini), and “Parkinson Institute Biobank” (Milan, http://www.parkinsonbiobank.com/) members of the Telethon Network of Genetic Biobanks funded by Telethon Italy, (http://www.biobanknetwork.org, project No. GTB12001)^47^.

The second set of human fibroblast lines (healthy control and G2019S diseased carriers) were obtained from the Coriell (NINDS) cell repository. Skin biopsies from which the fibroblasts were derived were collected under the informed consent protocols of Coriell/NINDS, established in culture and coded. Such biosamples obtained from the Coriell repository were not subject to a clinical intervention, nor did any of the researchers involved in the analyses have contact or any interaction with the biosample donors. The fibroblasts were grown in DMEM growth media supplemented with penicillin/streptomycin (1%) and FBS (10%). For each experiment, the cells were trypsinized, counted and re-seeded on 6 cm tissue culture plates or 96-well plates.

### Quantification of LRRK2 phosphorylation and in vitro kinase activity

To measure the in vitro kinase activity of LRRK2 purified from the different fibroblast lines, we employed an ELISA-based assay as described before^19^. Briefly, parallel ELISA plates were coated with anti-LRRK2 capture antibodies (Abcam; clone c41-2) overnight in coating buffer. The following morning, the plates were washed and 0.5 ug of fibroblast protein extract, diluted in TBST/1% BSA, was loaded into both plates (3 technical replicates, each) and incubated for 2 h at 37 °C under constant shaking. The plates were then washed 3× with wash buffer. On one plate, a detection antibody comprised of anti-LRRK2 (Abcam; clone UDD3) conjugated to HRP (Abcam; Lightning Link HRP conjugation kit) was added to each well and incubated for 1 h at room temperature, under constant shaking. In the second plate, we performed an in-well kinase reaction using biotinylated LRRKtide as a peptide substrate). The plates were washed an additional 2× in kinase buffer to equilibrate the wells, followed by addition of the kinase reaction mixture containing the peptide substrate and ATP (30 min at 30 °C). At the end of the reaction, the mixture was removed and saved, and the wells were washed a further 3× in ELISA wash buffer. In these wells, a detection antibody of anti-pS935 LRRK2 (Abcam; clone UDD2) was added for 1 h at room temperature under constant shaking. After both detection antibodies (total and pS935-LRRK2) were incubated for 1 h, the wells were washed again and ECL chemiluminescence substrate was added (ECL SuperSignal-Femto; ThermoScientific) for 5 min at room temperature. The signal was read using a Tecan Spark 10M microplate reader.

To quantify LRRK2 phosphorylation of the LRRKtide substrate, 0.5 μl of the kinase reaction containing biotinylated peptide was diluted into 50 μl of binding buffer, and added to streptavidin coated ELISA plates for 1 h at 37 °C, under constant shaking. After binding, the phosphorylated peptide was detected using anti-pThrxArg, followed by anti-rabbit HRP, each for 1 h at room temperature. Following each incubation, the wells were washed and finally ECL chemiluminescence substrate was added (ECL SuperSignal-Femto; ThermoScientific) for 5 min at room temperature. The signal from the total LRRK2 ELISA is then used to normalize the signal for pS935-LRRK2 and kinase activity, for each corresponding well.

### Cell lines, plasmids, and transfection

Human HEK293T cells and murine RAW264.7 cells control and Lrrk2 knockouts (ATCC), were grown in DMEM (Sigma; high glucose) supplemented with 10% FBS and penicillin/streptomycin. Primary fibroblasts, obtained from the Coriel Institute and from Istituto G. Gaslini^47^, were grown in DMEM plus 10% FBS and antibiotics. Plasmids encoding Flag-tagged wild type or mutant LRRK2 were previously described^48^. For transient transfection, HEK293T cells were incubated with DNA:CaPO_4_ precipitates or PEI as transfection reagents, and growth media was changed 24 h later.

### Crude lysosomal isolation

HEK293T or RAW264.7 cells were washed once with PBS, then collected in PBS and centrifuged at 3500 rpm for 5 min at 4 °C. The cell pellets were lysed in 0.25 M Sucrose/1×TBS buffer (pH 7.4) supplemented with 1× phosphatase inhibitors (Roche) in a manual glass homogenizer. The lysates were centrifuged at 6800 × *g* for 15 min at 4 °C. The supernatant was kept and the pellet was then washed with the same sucrose lysis buffer and centrifuged again as before. The combined supernatants from the previous steps were centrifuged at 21,000 × *g* for 30 min at 4 °C to yield a pellet containing lysosomes and supernatant containing cytoplasmic proteins. The lysosomal-enriched pellet was washed with the sucrose buffer and then resuspended in 50 μl activity assay buffer (50 mM citric acid, 176 mM K_2_HPO_4_, 10 mM sodium taurocholate, 0.01% Tween-20, pH 5.9). The lysosomal lysates were kept at −80 °C or were processed for analysis immediately.

### Electron microscopy of human fibroblasts

Samples were fixed with 2.5% glutaraldehyde in 0.1 M sodium cacodylate buffer pH 7.4 ON at 4 °C. The samples were postfixed with 1% osmium tetroxide plus potassium ferrocyanide 1% in 0.1 M sodium cacodylate buffer for 1 h at 4 °C. After three water washes, samples were dehydrated in a graded ethanol series and embedded in an epoxy resin (Sigma-Aldrich). Ultrathin sections (60–70 nm) were obtained with an Ultrotome V (LKB) ultramicrotome, counterstained with uranyl acetate and lead citrate and viewed with a Tecnai G2 (FEI) transmission electron microscope operating at 100 kV. Images were captured with a Veleta (Olympus Soft Imaging System) digital camera.

### Human differentiated dopaminergic neurons

Fibroblasts were isolated from skin biopsies of 4 control subjects with wild type *LRRK2*, 2 unrelated Parkinson’s disease patients carrying a heterozygous G2019S mutation of the *LRRK2* gene, 1 Parkinson’s disease patient carrying a heterozygous L444P mutation of *GBA1* gene, and 2 Parkinson’s disease patients carrying *SNCA* gene mutations (gene duplication and the heterozygous A53T mutation). Fibroblasts were reprogrammed into iPSCs through viral transfection of Klf4–Oct3/4–Sox2, cMyc, and Klf4 (CytoTune™-iPS 2.0 Sendai Reprogramming Kit, Thermo Fisher Scientific). One of the *LRRK2*-mutated iPSC line underwent genome editing to obtain an isogenic control with corrected *LRRK2* (LRRK2 #2 GC). In the same way, another control iPSC line (CTR5) was genome edited to generate a mutant G2019S *LRRK2* line (CTR5 G2019S). iPSCs were cultured in E8 medium (Thermo Fisher Scientific) and passaged with EDTA 5 mM every 1–3 days. Generated iPSCs underwent karyotype analysis, which was negative for chromosomal rearrangements. Proper reprogramming of iPSCs was assessed by expression of stem cell markers (SOX2, OCT4, TRA-1-60, SSEA4).

All iPSC lines were differentiated into dopaminergic neurons according to the protocol described by Kriks et al. (2011)^49^ with modifications as described in ref. ^26^. Immunofluorescent analysis for markers of dopaminergic identity (TUJ1, TH, and DAT) was performed to assess dopaminergic differentiation. Antibodies were used with following dilutions: TUJ1 (Abcam ab18207, 1:250), TH (Thermo Scientific PA5-17800 1:100), DAT (Millipore Ab2231, 1:600).

### LRRK2 transgenic animals

Homozygous LRRK2 G2019S (GSKI) and WT mice were housed at the National Institute on Aging, NIH, according to a protocol approved by the Institutional Animal Care and Use Committee of the National Institute on Aging, NIH (463-LNG-2019). Dissections of cortex, midbrain and striatal regions were performed in 6 months old mice of all genotypes. 4–5 mice/genotype were used in all experiments.

### Brain and fibroblast lysates preparation

Brain lysates were obtained by mechanically lysing the different regions on ice in 25 mM pH 7.5 Tris–HCl, 150 mM NaCl, 1% (v/v) NP40, 1% (w/v) sodium deoxycholate, 0,1% (w/v) SDS, 2 mM EGTA, 20 mM sodium fluoride, 50 mM beta glycerophosphate, 50 mM sodium pyrophosphate, 20 mM sodium orthovanadate. Fibroblasts were lysed in RIPA buffer, supplemented with protease inhibitors. Protein lysates were clarified by centrifugation and total protein concentration was determined by BCA assay (PierceTM, Thermo Fisher). Fifty micrograms of protein samples for brain lysates and 20 μg of protein samples were resolved on polyacrylamide gels (see Western Immunolotting for details).

### Measurement of GCase activity in vitro

The assay was performed in vitro using the fluorescent GCase substrates blue fluorogenic substrate 4-methylumbelliferyl-β-d-glucopyranoside (4-MU) (Sigma-Aldrich; as described in refs. ^11,12^). PBMCs were washed 2× with PBS and lysed in 60 μl of activity assay buffer (50 mM Citric acid, 176 mM K_2_HPO_4_, 10 mM sodium taurocholate, 0.01% Tween-20, pH 5.9) and we used for this assay a standard volume of 5 μl from total lysates. These total or lysosomal lysates were diluted in 5 μl activity assay buffer (see above) or 5 μl of 20 mM Conduritol B Epoxide (CBE) (Santa Cruz, sc-201356A) (specific GCase inhibitor) and incubated for 15 min at RT. Subsequently, 25 μl of 5 mM 4-MU (Sigma, M3633) were added and immediately incubated for 25 min at 37 °C. The reaction was stopped by adding 465 μl stop buffer (1 M NaOH, 1 M Glycine, pH 10). Fluorescence was measured (excitation 360 nm, emission 450 nm) in glass cuvettes in luminescence spectrometer (Perkin Elmer LS 55), given as relative fluorescence units (RFU). Each reaction was performed in duplicate. For each sample, the mean values in the presence or absence of the CBE inhibitor were calculated. All measurements were corrected by subtracting the average of nonspecific activity (incubation with CBE inhibitor) from specific average activity (incubation with activity assay buffer). The resulting measurements were then normalized to band intensities of GCase protein for lysosomal fractions of cell lines. For PBMCs the GCase activity measurements were first normalized to total protein concentration (Bradford method); or, as an alternative, to the band intensities of the GBA/GAPDH ratio following Western immunoblotting using ImageJ.

GCase activity was also measured in lysates from brain regions (midbrain, cortex, and striatum) of different transgenic mice, lysed as previously described, and in lysates from human fibroblasts. Brain or fibroblast lysates were diluted in McIlvaine buffer pH 4.5 (standard volume of 2–5 µl from total lysates was used), and a final concentration of 2 mM of the fluorogenic substrate 4MU (Sigma, M3633) were added to reach a final volume of 60 µl and immediately incubated for 90 min at 37 °C. The reaction was stopped by adding 200 μl of stop buffer (1 M NaOH, 1 M Glycine, pH 10). Fluorescence was measured (ex 360 nm, em 450 nm) in a plate reader (Victor X3, Perkin Elmer). Three technical replicates were performed for each experiment and all measurements were corrected by background subtraction. The resulting measurements were either normalized to band intensities of total GCase protein or by the total proteins in solution (and this is clearly stated in the results). A similar protocol was used to measure GCase activity in plasma from PD patients and healthy controls. In this experiment, the enzymatic activity assay was performed on 20 μl of plasma and normalized by total protein concentration as evaluated via BCA assay.

For GCase activity detection in dopaminergic neurons, 20 µg of proteins were incubated for 30 minutes at room temperature with 25 µl of McIlvaine buffer 4× (0.4 M citric acid, 0.8 M Na_2_HPO_4_) pH 5.2, AMP-DNM (N-(5-adamantane-1-yl-methoxy-pentyl)-deoxynojirimycin) at a final concentration of 5 nM, and H_2_O to a final volume of 100 µl. At the end of incubation, 25 µl of 4-MU was added at a final concentration of 6 mM and incubated for one hour at 37 °C. At the end of incubation, 10 µl of the reaction mixture were transferred to black 96-well plates and 190 µl of 0.25 M glycine pH 10.7 were added. Plates were read by Victor X3 microplate reader (Perkin Elmer). GCase activity was expressed as picomoles of converted substrate/mg of proteins/minute.

### In cell GCase activity measurement

The in cell GCase activity assay is based on the measurement of cellular fluorescence of the Fluorescein di-β-d-glucopyranoside (PFB-FDGlu) (Marker gene technologies) probe in a fluorescence microplate reader (Victor X3, Perkin Elmer). The substrate was used following the protocol reported by Benz et al. (2021). Briefly, cells were incubated 6 h with the PFB-FDGlu substrate, treatment with 100 μM CBE or with 100 nM Mli-2 were performed as previously described. Cells were then washed and lysed in RIPA buffer supplemented with protease inhibitors and PFB-FDGlu substrate fluorescence was measured as previously described. In both cases, to account for differences in cell seeding, signals were normalized to total protein concentration as evaluated using a BCA assay.

### Western immunoblotting

Equal amounts of protein were loaded on 10% SDS–PAGE gels. For brain lysates, 18-well Criterion SDS-gels were used (Cat# 1704273, Biorad). Primary antibodies were diluted in 5% milk/TBST or 5% BSA/TBST as recommended, and incubated with first antibodies overnight at 4 °C. The following primary antibodies were used: rabbit anti-LRRK2 (abcam, UDD3 ab170993, RRID:AB_2904228, 1:2000), rabbit anti-pS1292-LRRK2 (abcam, ab206035, RRID:AB_2904229, 1:2000), rabbit anti-pS935-LRRK2 [UDD2] (abcam, ab172382, RRID:AB_2904231, 1:1000), rabbit anti-LRRK2 (abcam, c41-2 ab133474, RRID: AB_2713963, 1:1000), rabbit anti-glucocerebrosidase (Sigma, G4171, RRID: AB_1078958, 1:1000), rabbit anti-Rab29 (abcam, ab199644, RRID:AB_2904232, 1:1000), rabbit anti-pT71-Rab29 (abcam, ab241062, RRID:AB_2884878), mouse anti-γ tubulin (Sigma, T5326-25UL, RRID:AB_532292,), mouse anti-GAPDH (Millipore, #MAB374, RRID:AB_2107445), rabbit anti-LIMP2 (Thermo Fisher Scientific, Cat#PA3-16802, RRID:AB_2182836, 1:1000), rabbit anti-SOD1 (Santa Cruz, sc11407, RRID:AB_2193779, 1:1000), rabbit anti-human LAMP2A (abcam, ab23322, 1:1000, RRID:AB_775981), mouse anti-LAMP1 (Santa Cruz Biotechnology Cat# sc-20011, RRID:AB_626853), rabbit anti-Rab10 (D36C4) antibody (Cell Signaling Technology Cat# 8127, RRID:AB_10828219, 1:1000), rabbit anti-RAB10 (phospho T73) antibody [MJF-R21] (Abcam Cat# ab230261, RRID:AB_2811274, 1:500), mouse anti-GBA antibody (Sigma-Aldrich Cat# WH0002629M1, RRID:AB_1841779, 1:1000), mouse anti-β-Actin Antibody (Sigma-Aldrich Cat# A5441, RRID:AB_476744). After washing 3 times for 10 min with TBST, the membranes were incubated with the appropriate secondary antibody conjugated with horseradish-peroxidase (HRP). Bands were visualized with chemiluminescence using Clarity ECL substrate (Biorad) or Immobilon Forte Western HRP Substrate (WBLUF0100, Merck-Millipore) as per manufacturer’s instructions.

### RNA extraction and qRT-PCR

RNA was extracted from 6-month-old frozen midbrains of WT and G2019S mice using Trizol/Chloroform extraction method as described by manufacturer (Invitrogen, Trizol reagent cat number #15596026).

RNA quality and concentration were assessed by Nanodrop. cDNA was synthesized from total RNA using SuperScript III First-Strand Synthesis SuperMix (Invitrogen, cat number 11752-050). 5 ng of cDNA have been used for each qRT-PCR reaction, that was carried in quadruplicates on 384-well plate. Additionally, 0.5 μl of each TaqMan probe Mm07298544_g1 for Ppid-VIC-MGB_PL and Mm00484700_m1 for GBA-FAM-MGB have been purchased from ThermoFisher Scientific and used simultaneously in the same reaction in addition to 5ul of TaqMan Fast Advanced Master Mix (Applied Biosystems, cat number #444552F3). The qRT-PCR assay was run on Applied Biosystems QuantStudio 6 Flex Real-Time PCR System and analyzed using Prism 9.

### Statistical analyses

All quantitative data are expressed as mean ± SEM (standard error of the mean) from at least 4 different mice/genotype or at least 3 independent cell cultures. Significance of differences between two groups was verified by Student t-test while comparisons between 3 or more groups were performed by one-way ANOVA with Dunnett’s Multiple comparison test/Bonferroni’s post-hoc test/Tukey’s post-hoc test.

## Supplementary information

Supplementary figure 1

Full Scan Gel Images

## Data Availability

The datasets generated during and/or analyzed during the current study are available from the corresponding author on reasonable request.
