# Supplementary material for: LRRK2 kinase activity regulates GCase level and enzymatic activity differently depending on cell type in Parkinson’s disease
Source: NPJ Parkinsons Dis. 2022 Jul 19;8:92. doi: 10.1038/s41531-022-00354-3 (PMC9296523; doi:10.1038/s41531-022-00354-3)
Supplement: Supplementary file 1 — Supplementary figure 1 [file 41531_2022_354_MOESM1_ESM.pdf]

LRRK2 kinase activity regulates GCase level and enzymatic activity differently depending on cell type in Parkinson's disease

Maria Kedariti[1], Emanuele Frattini[2,3], Pascale Baden[4], Susanna Cogo[5,10], Laura Civiero[5,11,12], Elena Ziviani[5], Gianluca Zilio[5], Federico Bertoli[4], Massimo Aureli[6], Alice Kaganovich[5,7], Mark R Cookson[7], Leonidas Stefanis[1,8], Matthew Surface[9], Michela Deleidi[4], Alessio Di Fonzo[2,3], Roy N. Alcalay[9], Hardy Rideout[1,CA], Elisa Greggio[5,12,CA] and Nicoletta Plotegher[5,CA]

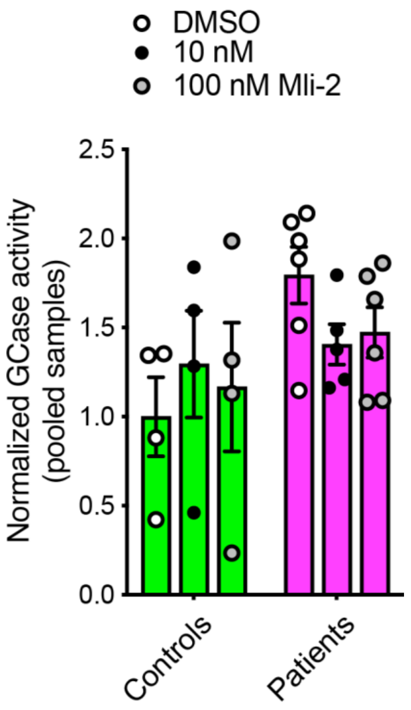

Supplementary Figure 1

GCase activity assessed with the in-cell method from Benz 2021 protocol. No differences were observed between genotypes or treatments (two-way ANOVA, Tukey's multiple comparisons test). Each dot represents one subject and the value is the average of 2 biological replicates each with 3 technical replicates for DMSO and one biological replicate with 3 technical replicates for 10 nM and 100 nM MLi-2.
