# Supplementary material for: LRRK2 kinase activity regulates GCase level and enzymatic activity differently depending on cell type in Parkinson’s disease
Source: NPJ Parkinsons Dis. 2022 Jul 19;8:92. doi: 10.1038/s41531-022-00354-3 (PMC9296523; doi:10.1038/s41531-022-00354-3)
Supplement: Supplementary file 2 — Full Scan Gel Images [file 41531_2022_354_MOESM2_ESM.pdf]

Midbrain mice lysates (Fig. 1b)

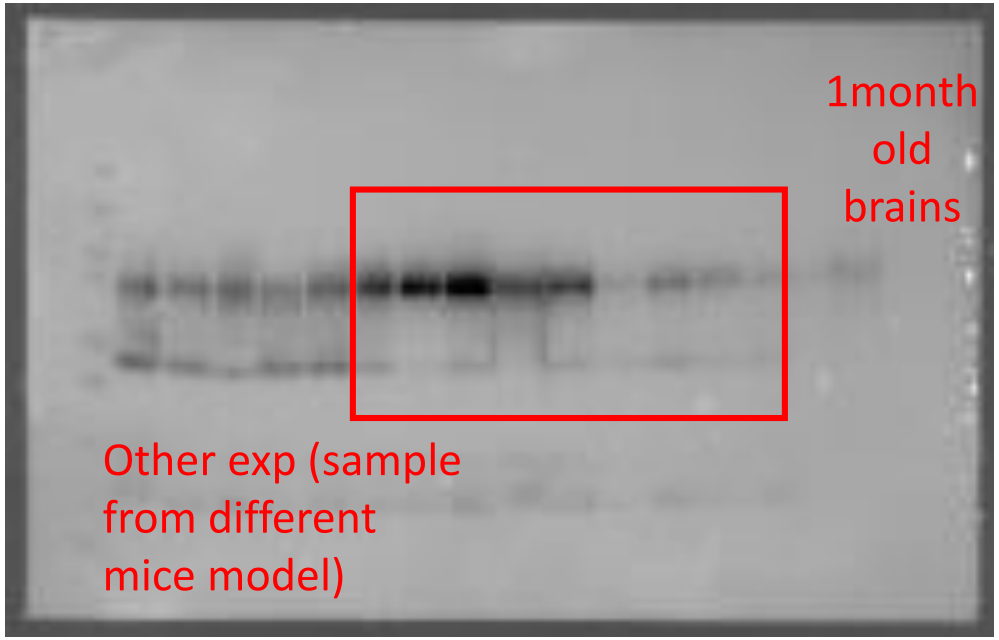

IB: GBA

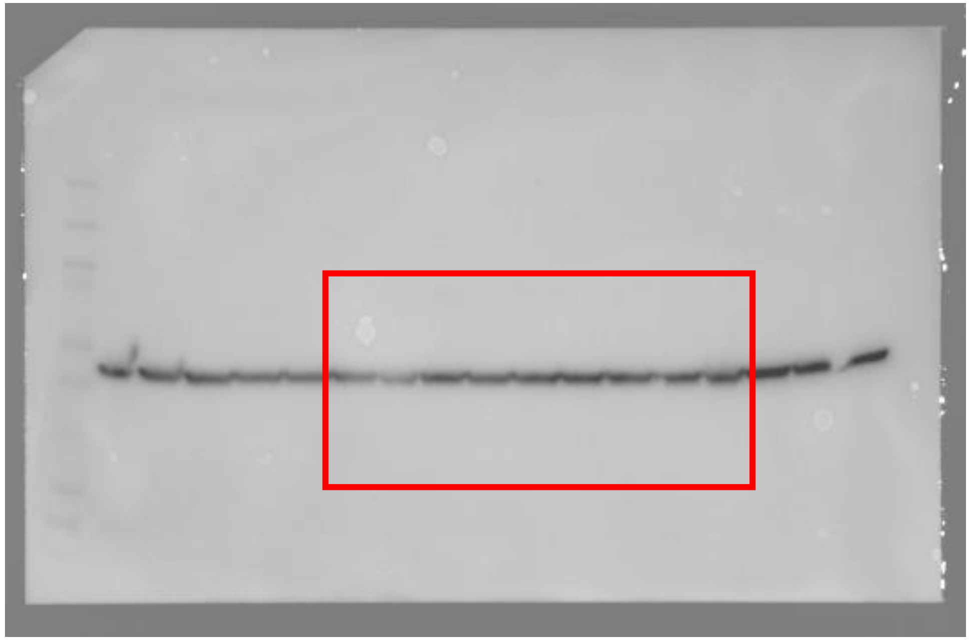

IB: actin

Human PBMCs (Fig. 2b)

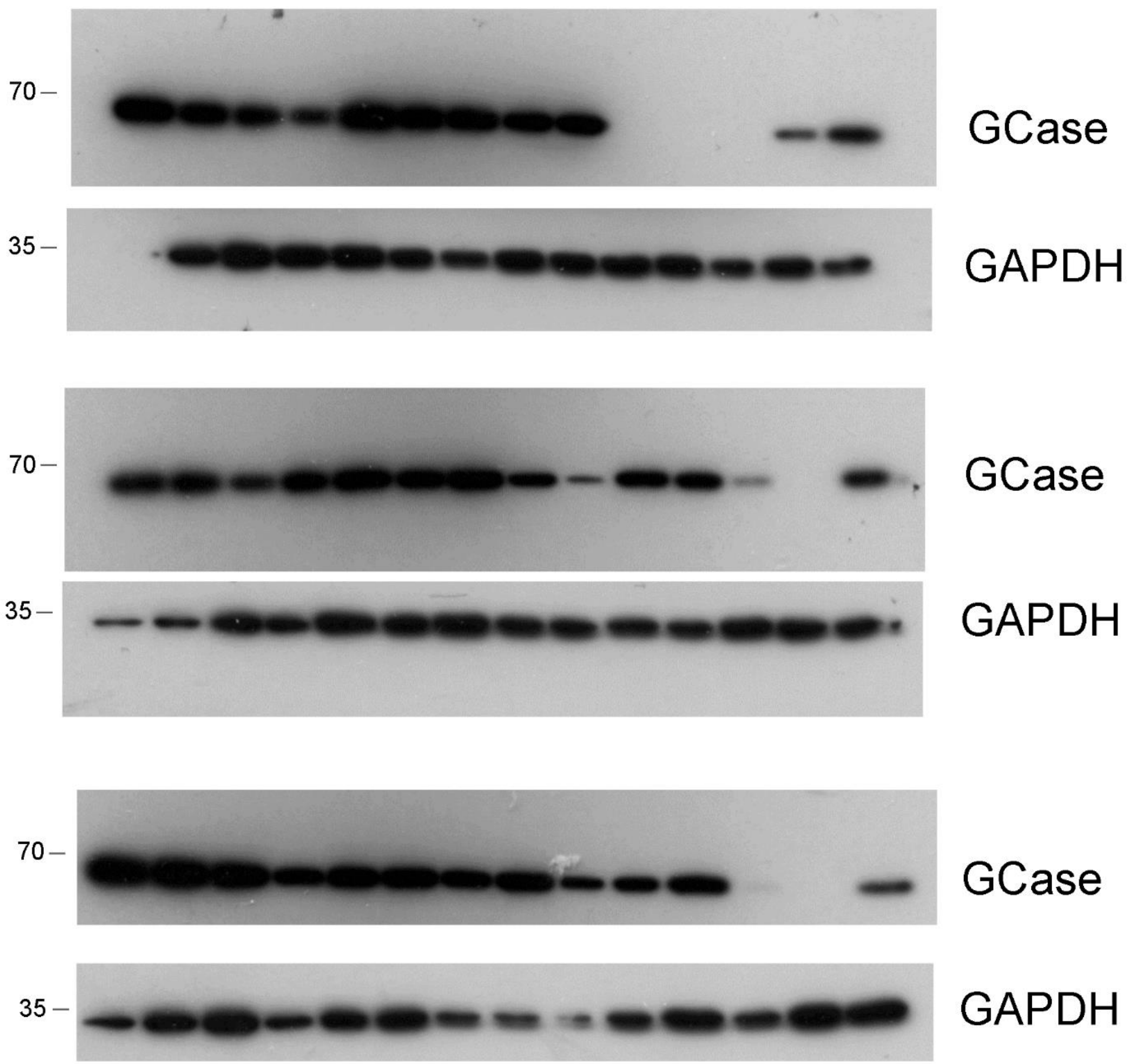

A. Human Fibroblasts (Fig. 3d)

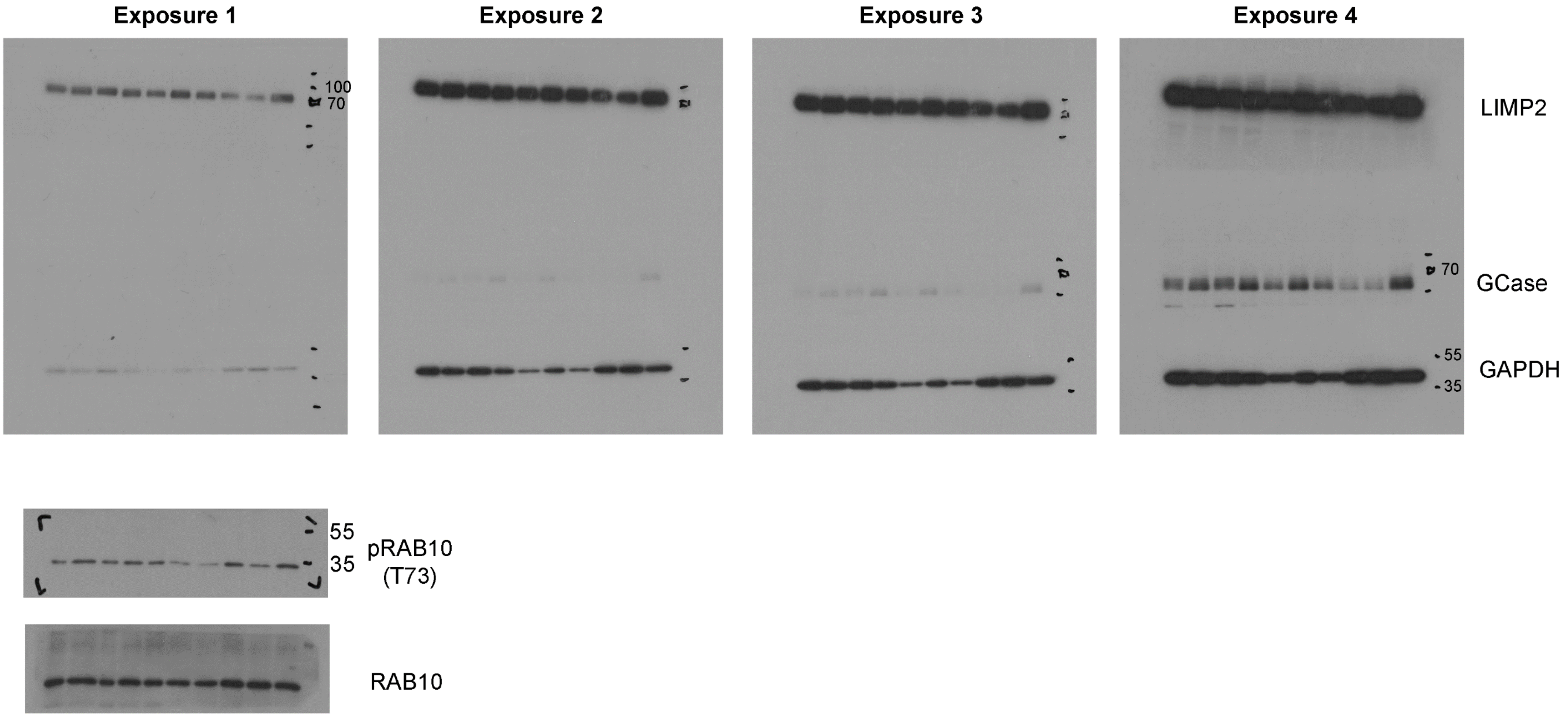

iPSC-derived neurons lysates (Fig. 4e)

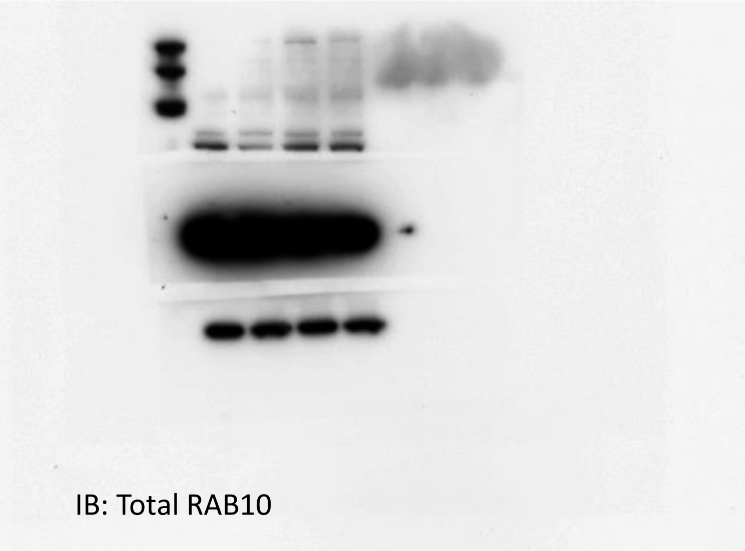

IB: Total RAB10

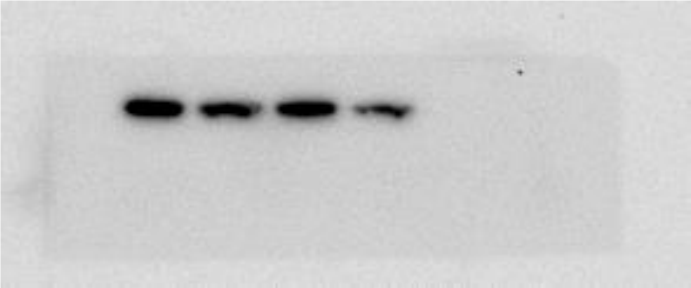

IB: pRAB10

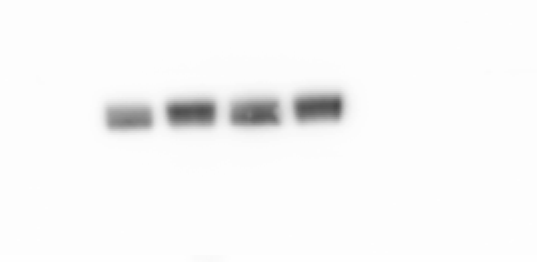

IB: GBA

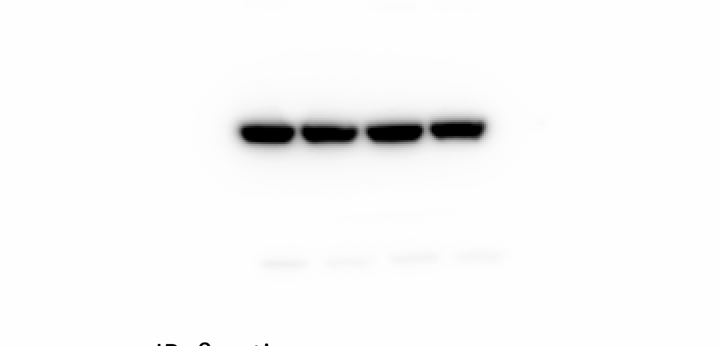

IB: β-actin

Figure 5a: HEK293T overexpressing LRRK2 and treated with MLi-2

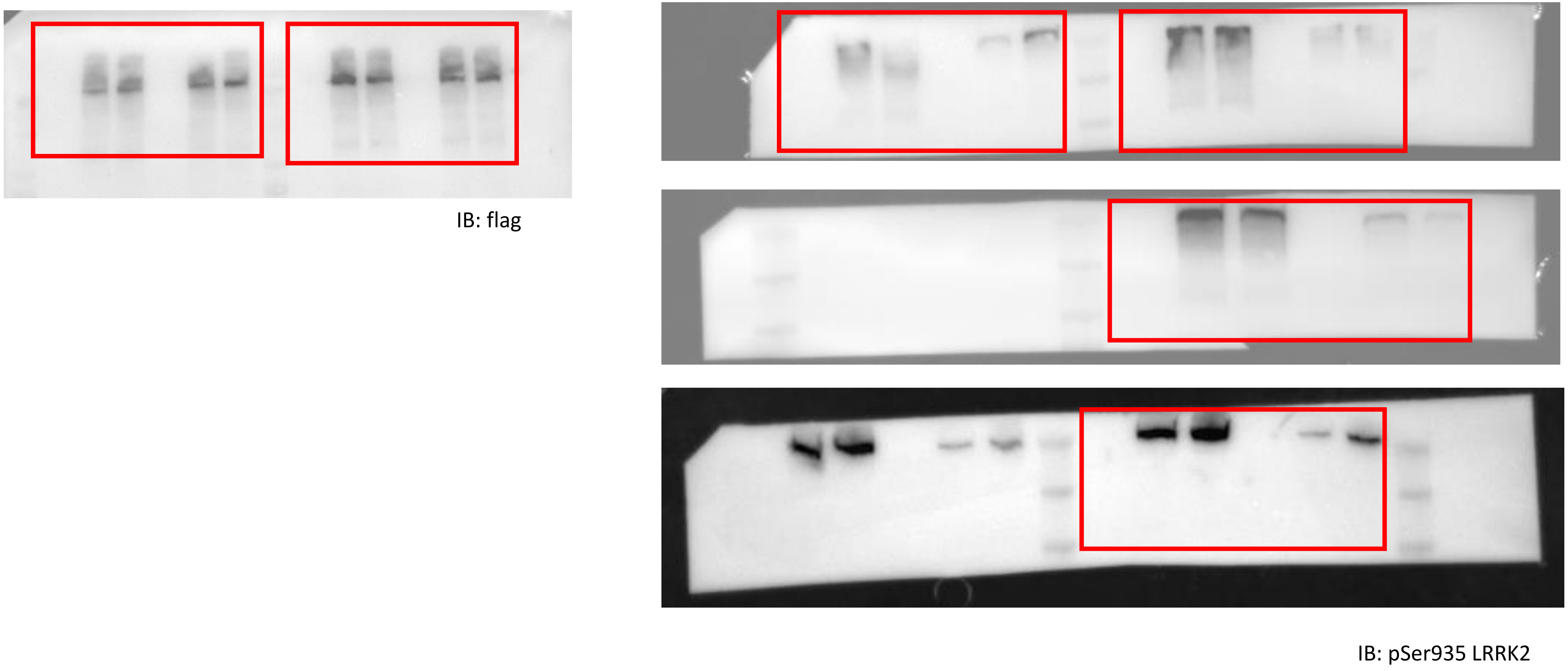

All blots were loaded in the same order as in the manuscript

Figure 5a: HEK293T overexpressing LRRK2 and treated with MLi-2 (GBA and actin)

IB: GBA

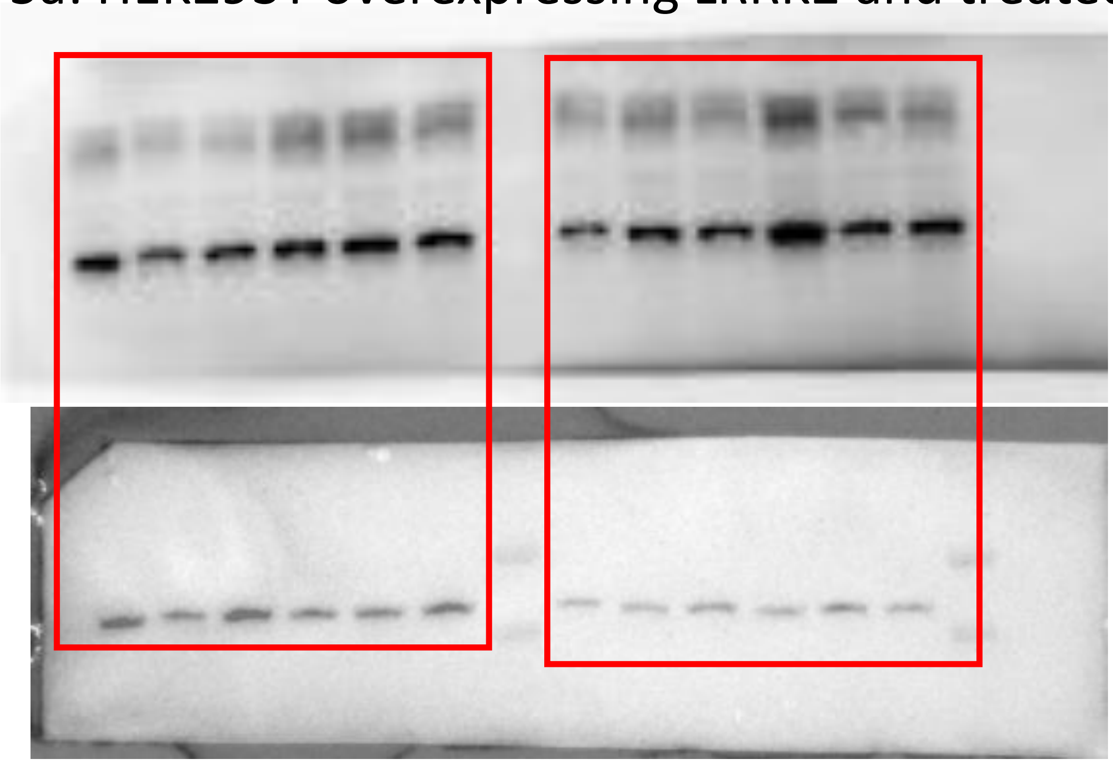

IB: β-actin

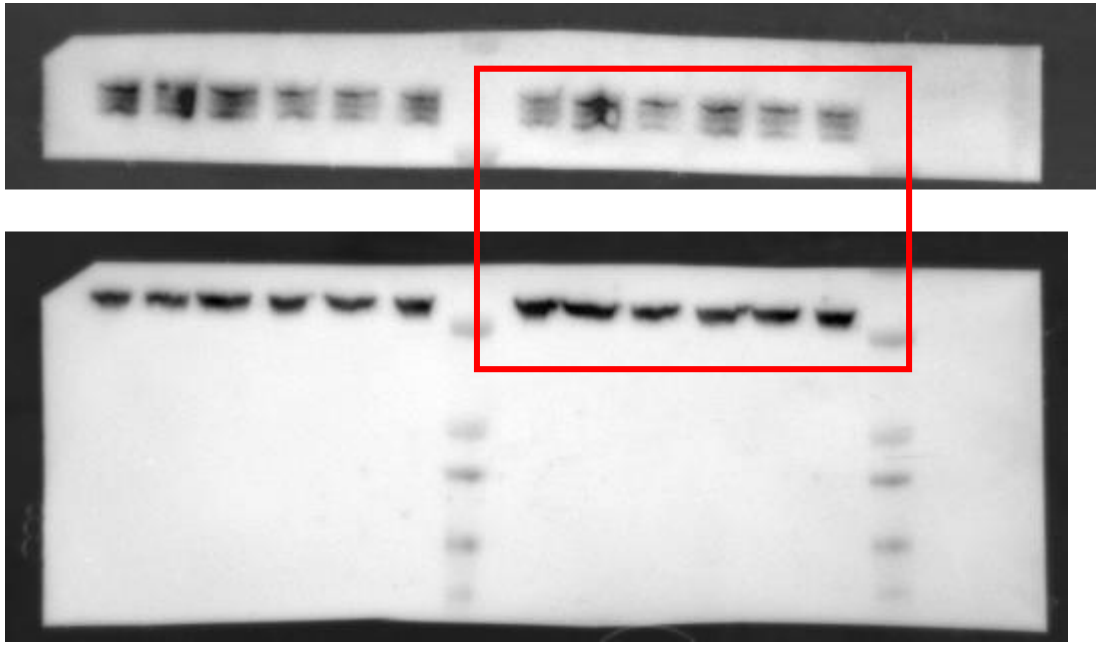

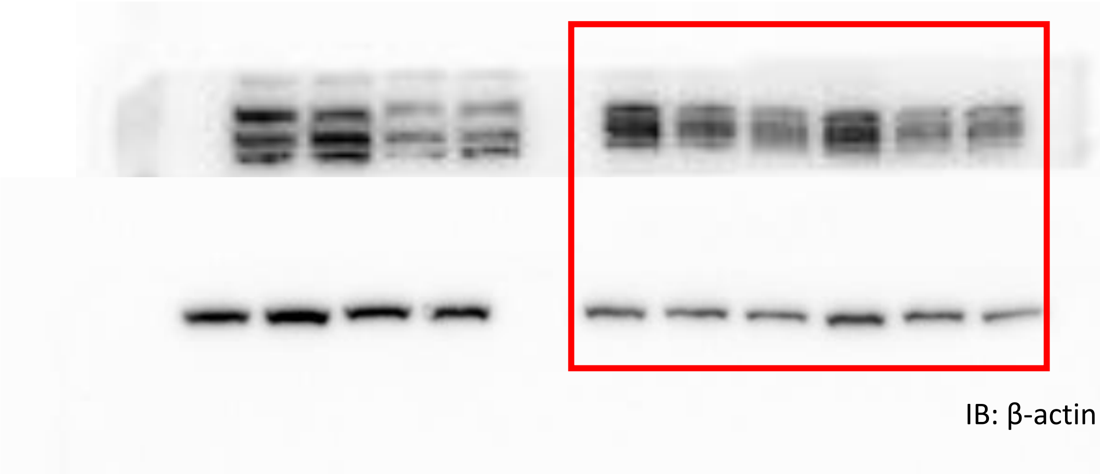

IB: GBA

IB: β-actin

All blots were loaded in the same order as in the manusript

Human HEK293T (Fig. 5c; whole cell extracts)

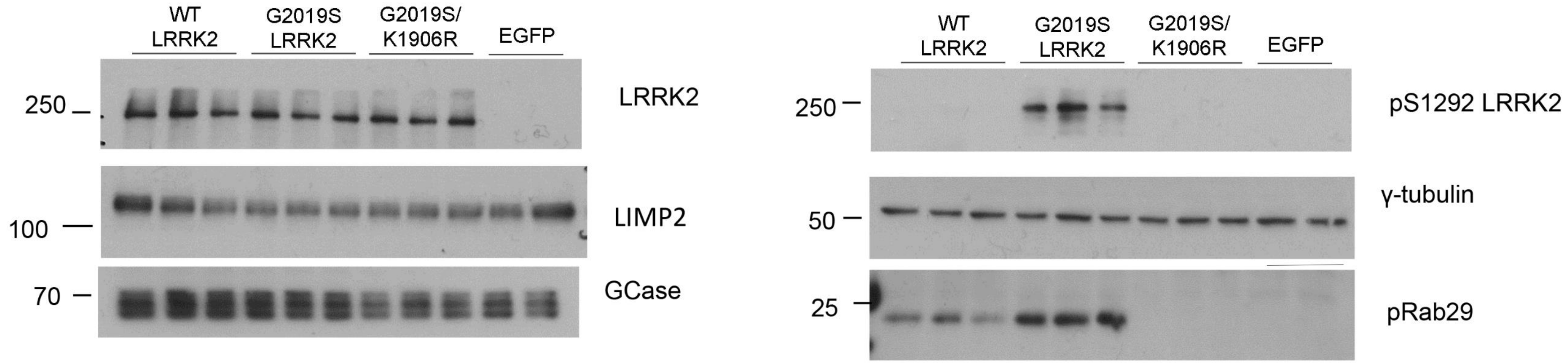

Human HEK293T cells (Fig. 5d)

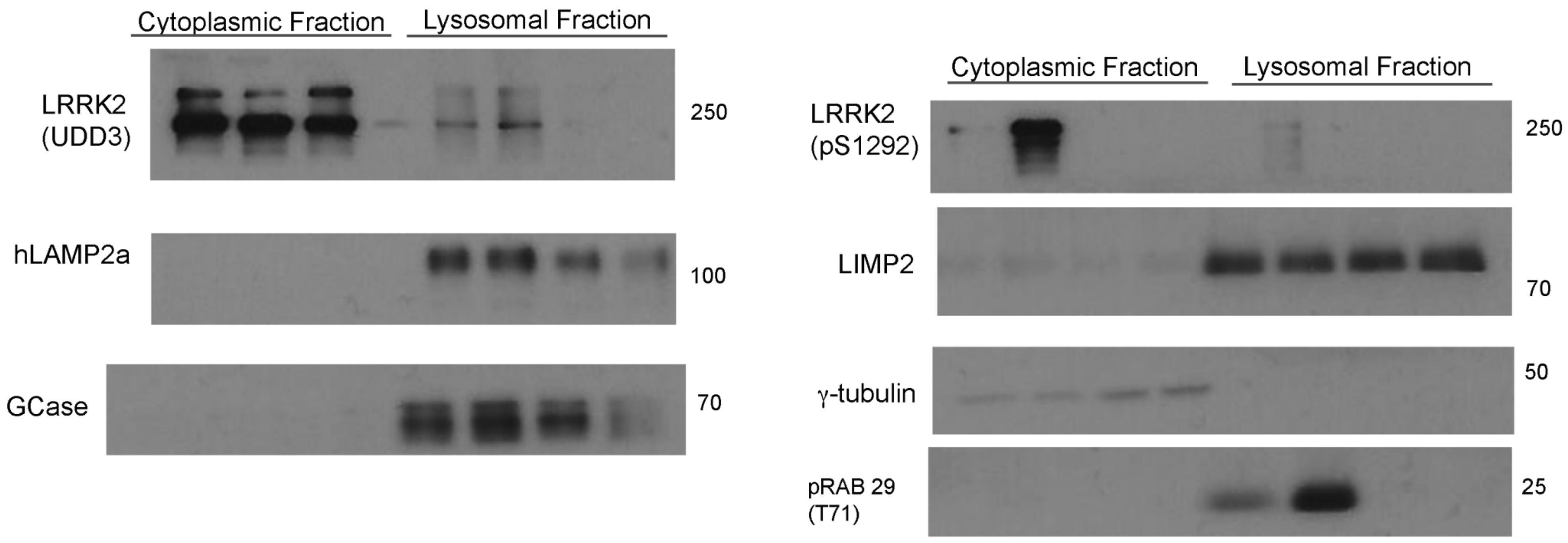

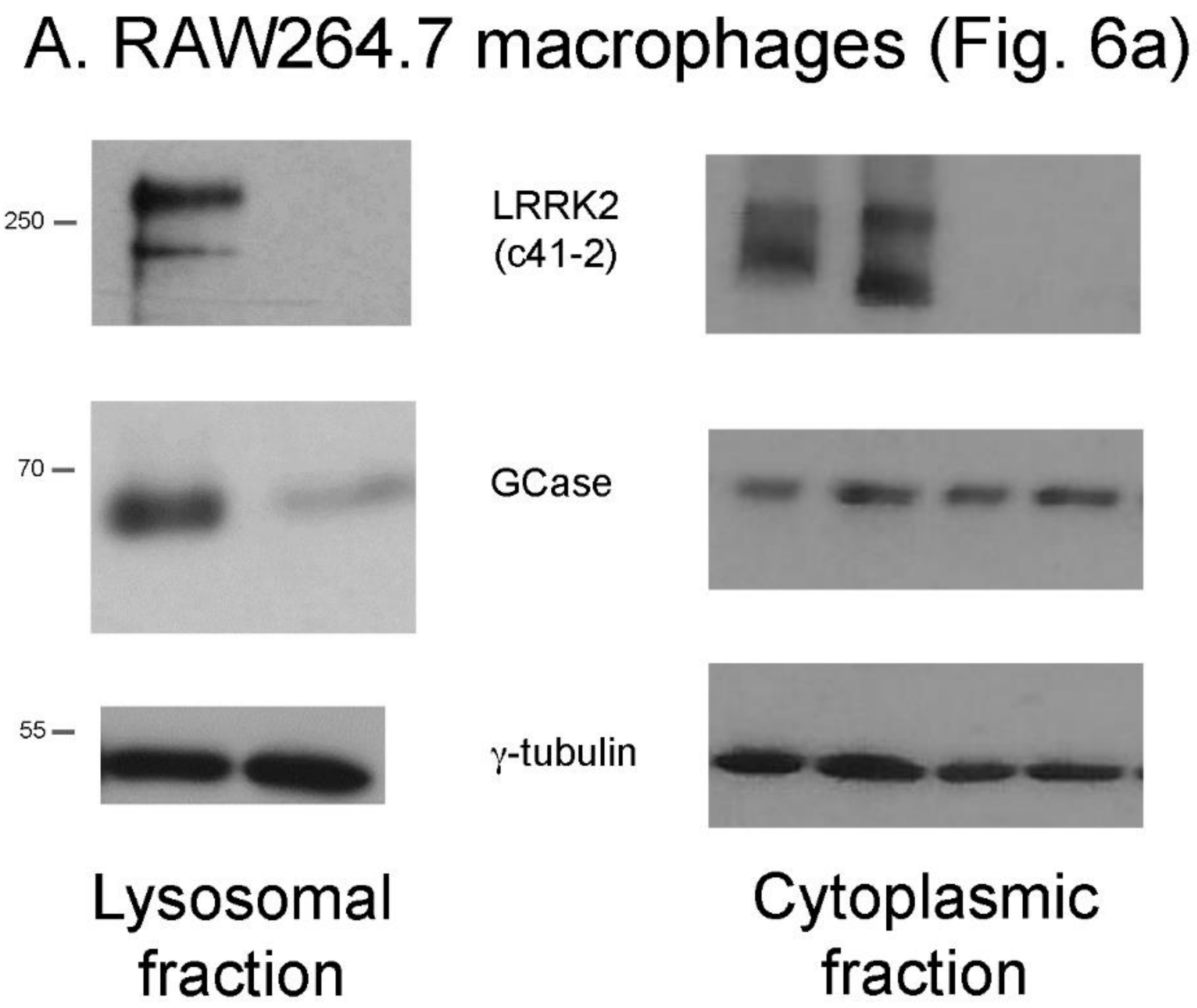
A. RAW264.7 macrophages (Fig. 6a)
250
LRRK2
(c41-2)
70
GCase
55
γ-tubulin
Lysosomal
fraction
Cytoplasmic
fraction

Raw WT and KO LRRK2 (Fig. 6b)

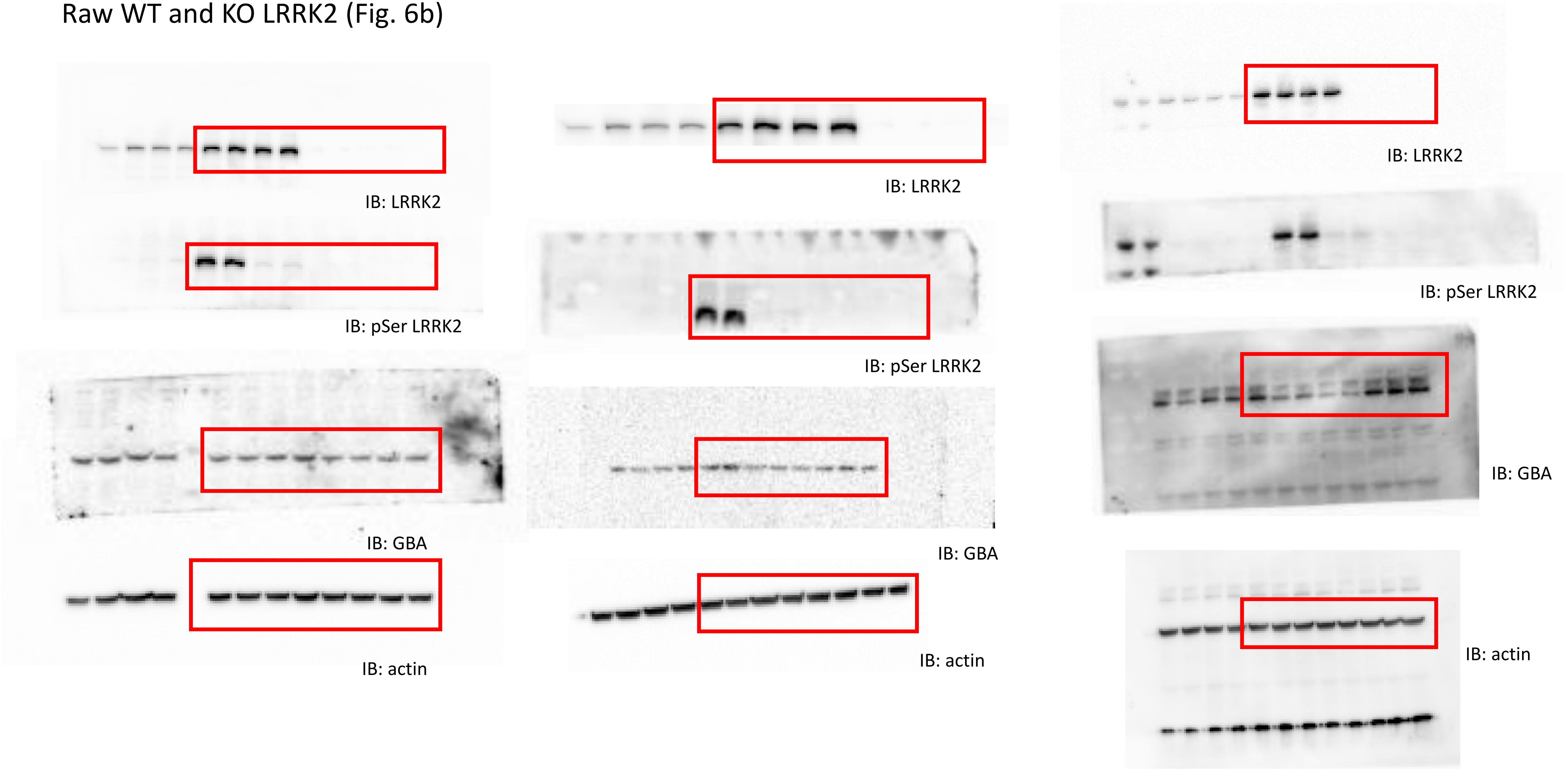

All blots were loaded in the same order as in the manuscript

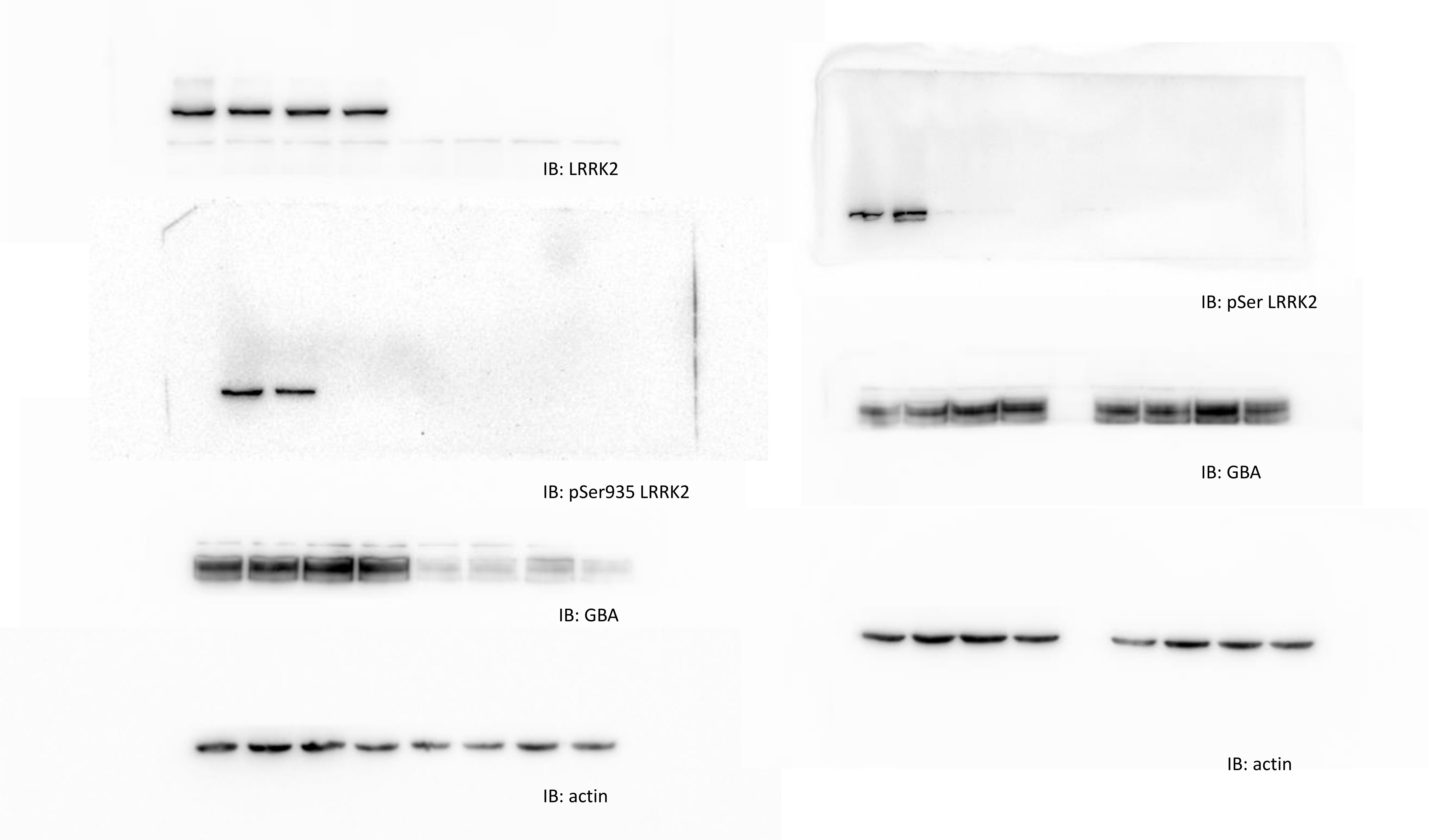

All blots were loaded in the same order
